# Area level indirect exposure to extended conflicts and early childhood anthropometric outcomes in India: a repeat cross-sectional analysis

**DOI:** 10.1186/s13031-023-00519-8

**Published:** 2023-05-07

**Authors:** Pritha Chatterjee, Jarvis Chen, Aisha Yousafzai, Ichiro Kawachi, S. V. Subramanian

**Affiliations:** 1 Department of Social and Behavioral Sciences, Harvard T.H. Chan School of Public Health, 677 Huntington Avenue, Boston, MA 02115 USA; 2 Department of Global Health and Population, Harvard T.H. Chan School of Public Health, 677 Huntington Avenue, Boston, MA 02115 USA; 3 Harvard Center for Population and Development Studies, Cambridge, MA 02138 USA

**Keywords:** Child growth, Child anthropometry, Violence, Conflicts, India

## Abstract

**Background:**

Protracted, internal conflicts with geographic variations within countries, are an important understudied community exposure for adverse child health outcomes.

**Methods:**

Violent events from the Uppsala Conflict Data Program (UCDP) between January 2016–December 2020 and January 2010–December 2015, were included as exposure events for children sampled in National Family Health Surveys (NFHS) 5 (2019–21) and NFHS 4 (2015–16), respectively. Geocoded data from UCDP were merged with residential clusters from NFHS, to identify children living in villages or urban blocks situated at <= 50 km from conflict sites. Within these clusters, which we defined as conflict exposed, we studied risks of stunting, underweight and wasting in children, prenatally, and in 0–3 years. We assessed sensitivity on a subsample of siblings with discordant conflict exposures.

**Results:**

For NFHS 5, exposure to violence between 0 and 3 years was associated with 1.16 times (95% CI 1.11–1.20) higher risks of stunting, 1.08 (1.04, 1.12) times higher risks of underweight, and no change in wasting. In-utero violence exposure was associated with 1.11 times (95% CI 1.04–1.17) higher risks of stunting, 1.08 (95% CI 1.02–1.14) times higher risks of underweight, and no change in wasting, among children <= 2 years. In 17,760 siblings of 8333 mothers, exposure to violence during 0–3 years, was associated with a 1.19 times higher risk of stunting (95% CI − 0.24 to 0.084). Incremental quartiles of violence exposure had higher risks of stunting and underweight until quartile 3.

**Conclusion:**

In-utero and early childhood indirect exposure to protracted conflicts were associated with increased stunting and underweight in India. Given the continued exposures of such historically and contextually rooted internal conflicts in many LMICs, chronic violence exposures should be targeted in public health policies as important social and political determinant of child health.

**Supplementary Information:**

The online version contains supplementary material available at 10.1186/s13031-023-00519-8.

## Introduction

Over 1.6 billion children live under conflict conditions, 426 million within 50 km (km) of conflict events. While area level, indirect conflict exposures in Low-And-Middle-Income-Countries (LMICs) are studied in their association with children’s health widely [1], much of this evidence is focused on acute, cross-border events [2]. Protracted internal conflicts are long standing struggles rooted in local histories, politics and inter social, religious and linguistic group relationships that affect many LMICs [3]. They are understudied particularly in their associations with longer term outcomes beyond immediate threats to child mortality and health system disruptions [4–6].

The International Committee of the Red Cross defines violent events extending for eight or more successive years as protracted conflicts [3, 7]. The Lancet Bridging Research & Action in Conflict Settings (LBRACS) series has termed conflict epicenters as areas of active warfare, and insecure areas as those with histories of‚ and potential for future violence [8]. Chronic conflicts, characteristic of “insecure areas” in the LBRACS’ classification, have been observed in varying types, lengths, intensities and proximities of influence on children [9–11]. Children’s exposure to such conflicts may vary within countries geographically, and by their social group identities [7, 9]. Such conflicts may have distinct pathways of influence, and be associated with unique adaptive bioregulatory mechanisms [9, 12]. Additionally, as Cummings et al. [6, 13] have posited, sectarian or politically motivated violence may have unique outcomes and mechanistic pathways related to children’s emotional insecurity, compared to other forms of community violence. Moreover, children of minority social groups may be especially vulnerable to sectarian violence, compared to non-sectarian violence [13, 14].

Living in proximity to conflicts, characterized as indirect conflict exposures, may cause adverse outcomes in children [2, 10, 15]. From a measurement perspective, such area level exposures are typically accounted for, by administrative boundaries like districts [6]. However, smaller geographies may be more meaningful for lower intensity and/or acute events, whereas larger scale, longer episodes of violence may have wider scopes of influence [16, 17]. Often, ranges of 50–100 km are set to define exposures to large scale conflicts [5, 10, 18]. While such distance measures have been accepted as decent estimates of children’s indirect exposure to conflict, there is limited understanding of how distance from sites of conflict, and variations in their intensity influence child health outcomes [10]. Additionally, physical distance may not be meaningful with spread of threats from news and social media [19]. Moreover, in the case of sectarian violence, children from specific social groups may be especially vulnerable to violence exposure, due to their “emotional proximity” for violent events targeted at their communities [13]. Additionally, female children have been found to have more deleterious outcomes for both physical and socioemotional outcomes, in cases of direct and indirect conflict exposure [20]. Frequency and timing of exposure in the child’s life are also understudied aspects of conflict exposures, that may modify the effects of physical distance from site of conflict [9, 21, 22].

The developmental period of conflict exposure is important to child health outcomes under study [22]. Perinatal and early childhood conflict exposures influence outcomes in infancy, childhood and adolescence [6, 13, 22, 23]. Exposures between 0 and 2 years are associated with poor Height-For-Age-Z-scores (HAZ) in children in Rwanda, Nigeria, Ethiopia, Burundi, Cote d’Ivoire and Iraq [24–28].Multiple pathways have been hypothesized for poor stature, including reduced food supply, interruption in health services, and accumulated allostatic load [10, 28]. Conflicts may also precipitate infectious and communicable diseases from displacement, crowding and sub-par sanitation, leading to poor child growth [2, 6]. Furthermore, studies from Syria, Timor-l’Este, Uganda, and Lebanon suggest that conflicts may trigger parental distress [29, 30], which has deleterious effects on caregiving practices like early initiation of and continued breastfeeding, which are in turn correlated with child growth [29–31]. Evidence from Pakistan and Sierra Leone suggests disruption in immunization under conflicts could lead to poor child growth [6, 20, 32]. In-utero conflict exposures cause lower utilization of perinatal services [16, 33, 34], and decreased institutional births, which in turn have been linked to lower birth weight [26]. Lower birth weight is associated with higher risks of stunting and reduced cognitive function in adolescence [25, 35, 36]. Early childhood conflict exposures are also associated with chronic inflammation, diarrheal disease, malnutrition, and changes in brain morphology including reduced brain volume, and activation of resting state networks [22, 37, 38].

Across this literature, extended conflicts remain understudied in their association with outcomes other than child mortality [2]. Moreover, among nutritional outcomes, child HAZ has been studied more extensively than Weight-for-Age-Z-scores (WAZ) and Weight-for-Height-Z-scores (WHZ), which are reflective of more short term nutritional disruptions [6, 10]. Additionally, the timing of conflict exposures in relation to children’s developmental periods has been examined only in limited studies [2, 39]. Finally, despite an extensive literature studying conflicts as a social and political determinant of child health, quantitative evidence from India on conflicts and children’s growth and overall physical health is sparse [4]. With multiple social, religious and linguistic groups and a history of different types of extended conflicts [4, 40], India also contributes to a third of stunted and half of wasted children, globally [41, 42].

To address these gaps, we examined the association of different types of extended community violence events in India with indicators of short- and long-term child growth, for two different child development periods- in-utero and early childhood exposures. While using a distance measure to define our conflict exposure, we assessed the role of increasing distance from sites of conflicts, conflict intensity, and chronicity, following the approach of Wagner et al. [43–45] in studies of political conflicts in the African continent. In line with Cumming et al.’s [13] recommendations of the effects of sectarian violence on minority children, we also studied effect measure modification patterns in associations of conflict exposure and child health by child’s caste and religious identities, as well as by other individual level socioeconomic characteristics, and sex. To build hypotheses about mechanisms, we studied how conflicts were associated with childhood anemia and probability of low birth weight, as two indicators of nutritional disruptions, that may be associated with growth outcomes.

## Methods

*Data* We used two waves of the nationally representative National Family Health Survey (NHFS) for 2015–16 (NFHS 4) and 2019–21 (NFHS 5). NFHS 5 collected height and weight measures for 235,155 children aged 0–5 years, retrospective birth history for 724,115 women in 29,777 geo-referenced villages, and urban blocks which served as the primary sampling units (PSUs) [46]. Data collection for NFHS 5 was conducted in two phases (phase one from June 17, 2019 to January 30, 2020 and phase two from 2 January 2020 to 30 April 2021), with a halt between April and October 2020, due to the COVID-19 lockdown [47]. NFHS 4 collected data on 264,049 children aged 0–5 years, and retrospective birth history for 699,686 women in 28,000 geo-referenced villages and urban blocks, between December 2015 and January 2016 [46].

Data on protracted conflicts was obtained from the Uppsala Conflict Data Program (UCDP), which collates data on geo-referenced conflict events, the actors involved, time, location, and reported deaths from secondary sources [48].This data source has been used for studies measuring exposure to conflict as a determinant of children’s and women’s health by different studies in LMICs [26, 43–45, 49]. Importantly, UCDP only includes violent events causing >= 25 deaths annually [48]. In India, this captures conflict events from the Jammu and Kashmir (J&K) conflict in the north, the Naxal-Maoist insurgency in western, central and eastern states, insurgencies in the north-east, and violence from Hindu-Muslim riots, all extended conflicts which have been occurring since India’s independence in 1947 [48]. Importantly, UCDP omits other important extended conflicts, including caste related violence, violence against Muslims and other minorities [40, 50, 51].

*Analytic sample* Latitude, and longitude identifiers of conflict sites from UCDP data were matched with geocodes of sampled children’s residential clusters in NFHS, to measure Haversine distance [52]. Based on Wagner et al.’s [43–45] approach for identifying exposure to high intensity conflicts in the African continent, NFHS clusters situated within 50 km from conflict sites were defined as conflict exposed [10]. As discussed in the sensitivity analysis section, we also validate this by using different distance cut-offs for conflict exposure.Conflict events captured in the UCDP data were geographically patterned by historical geopolitical secessionist movements [53]. Therefore, to minimize confounding by state and geography, we restricted our analysis to residential clusters we defined as conflict exposed. We then constructed two repeat cross-sectional analytic samples. Conflict events that began and concluded between June 2014–December 2020, and January 2010–September 2016 were each included as exposure events for children sampled in NFHS 5 (2019–21) and NFHS 4 (2015–16). Only conflict events that started >= 90 days from the date of measurement of children’s height and weight were included to allow for temporal precedence of conflict exposure.

*Exposure* Conflict exposure was defined using a binary specification (exposed to conflicts and not exposed to conflicts). Additionally, distributions of numbers of conflicts and conflict-related deaths experienced by each child were used to define quartiles of conflict chronicity and intensity, respectively. In both cases, children who experienced no violence constituted the reference population. For both specifications of the exposure, we studied conflict exposure in-utero (from conception to birth) and in early childhood (between 0 and 3 years), in children who were <= 2 years, and <= 5 years respectively, when they were measured for height and weight (Additional file 1: Fig. S6). These together comprise the first 1000 days which are a critical developmental period for nutrition, growth and brain development [23]. We also assessed outcomes of children who experienced conflicts in both developmental periods.

*Outcomes* We studied anthropometric outcomes defined as per WHO child reference standards of z-scores <=  − 2 for height-for-age (HAZ, stunting), weight-for-age (WAZ, underweight) and weight-for-height (WHZ, wasting) [54]. Absolute values of above 6 in these values were specified as missing. Stunting is considered a marker of accumulated adverse exposures and chronic undernutrition, which is also indicative of long-term developmental potential [54]. Wasting is associated with short-term, acute disruptions in nutrition specific and sensitive interventions [55]. Underweight captures both short- and long-term disruptions and may be influenced by both stunting and wasting [54].

*Covariates* Variables associated with conflict exposure and child anthropometry in the literature were included as covariates, including child’s age, sex, caste, religion, household wealth quintile, state of residence, urbanicity, maternal education, maternal height, or weight. Caste and tribal identities were classified as Schedule Castes and Schedule Tribes, Other Backward Castes, and the reference category of other castes, or Forward Castes, who constitute the most privileged group. Religious groups including Muslims, Christians, and Sikhs, Jains, Buddhists, were collectively classified as “Other” religions due to their smaller numbers. Hindus, the majority religion was specified as the reference group. The household wealth index was constructed by Principal Component Analysis of measures of living standards and asset ownership [46]. Maternal education was classified as no schooling, primary, secondary, higher secondary schooling, and college education or above. Mother’s height was categorized as < 145, 145–149.9, 150–154.9, 155–159.9, and 160 + cm.

*Estimation strategy* We estimated the risks of stunting, underweight and wasting associated with experiencing conflicts during each developmental period, within clusters we defined as conflict exposed. Children living in conflict exposed clusters who did not experience any violence comprised the counterfactual population, following the identification strategy of Wagner et al. [43–45] in their work on Africa associations of conflict exposure and child and women’s health outcomes. As the prevalence of each outcome was above the 10% threshold of a rare outcome [56], in our statistical modeling approach, we specified Log Poisson regressions. We also included state fixed effects to control for time invariant state level factors, as well as the child’s age to control for common time varying factors that applied across states. Heteroscedasticity-robust standard errors corrected for clustering at the state level were estimated. Finally, effect modification patterns were studied for child’s sex, religion, caste, household wealth, mother’s education, and types of violent events. We present two sets of cross-sectional analyses for children sampled in NFHS 5 (2019–21) and NFHS 4 (2015–16).

*Sensitivity analyses* First, to minimize risks of confounding from unmeasured mother level variables, we restricted our analysis to a subsample of siblings who were discordant in their exposures to conflicts, with fixed effects for mothers. Second, we assessed how increasing distance from site of conflict influenced estimates, using a variable for logarithm of the distance measure in kilometers. We also used distance cut offs of 75, 100, and 200 km from sites of conflict to check sensitivity to different definitions of conflict exposed clusters. Third, to minimize risks of biases from COVID-19 and its lockdown, we did three sensitivity checks on the sample of children sampled in NFHS 5. Data collection for NFHS 5 was carried out in two phases- pre and post Covid related lockdowns, in 2019–2020 and in 2021, respectively [47]. Based on the dates on which children were measured for their height and weight, we assessed possible confounding from the COVID-19 induced lockdown (Additional file 1: Table S1.1). In 22 states/union territories, children were almost entirely (> 92%) sampled prior to the lockdown, and in 13 states/UTs, children were sampled across both phases, with 25–73% children being sampled pre lockdown (Additional file 1: Tables S1.2, S1.3). We compared prevalence of anthropometric outcomes in children who were sampled in pre and post COVID-19 related lockdown phases (Additional file 1: Table S1.4). ﻿Next, we restricted the analysis to the subsample of states where majority of children were sampled before the lockdown, with the hypothesis that these states would be less prone to biases from the pandemic. Finally, we restricted the sample to the 13 states where children were sampled in both phases, with a child level binary variable to indicate whether sampling occurred pre or post COVID-19 lockdown. As secondary outcomes, we studied how exposure to protracted conflicts was associated with birth weight in children  <= 2 years; and with childhood anemia for children exposed between 0 and 3 years, after controlling for maternal anemia. Childhood anemia was defined as hemoglobin < 11 g/dl. Since maternal anemia was a significant predictor of childhood anemia, we also studied how violence exposure during the last two years before the survey was associated with mothers’ anemia level [57, 58].

*Patient and Public Involvement* Since the study was based on de-identified survey data, participants were not involved in the design. However, the research question and hypotheses were formed by multiple press and human rights reports on violence and children’s exposure to violence in LMICs. Findings from this research will be used to publish op-eds to highlight conflicts as important political determinants of child health.

## Results

### Descriptive statistics

**Fig. 1:**
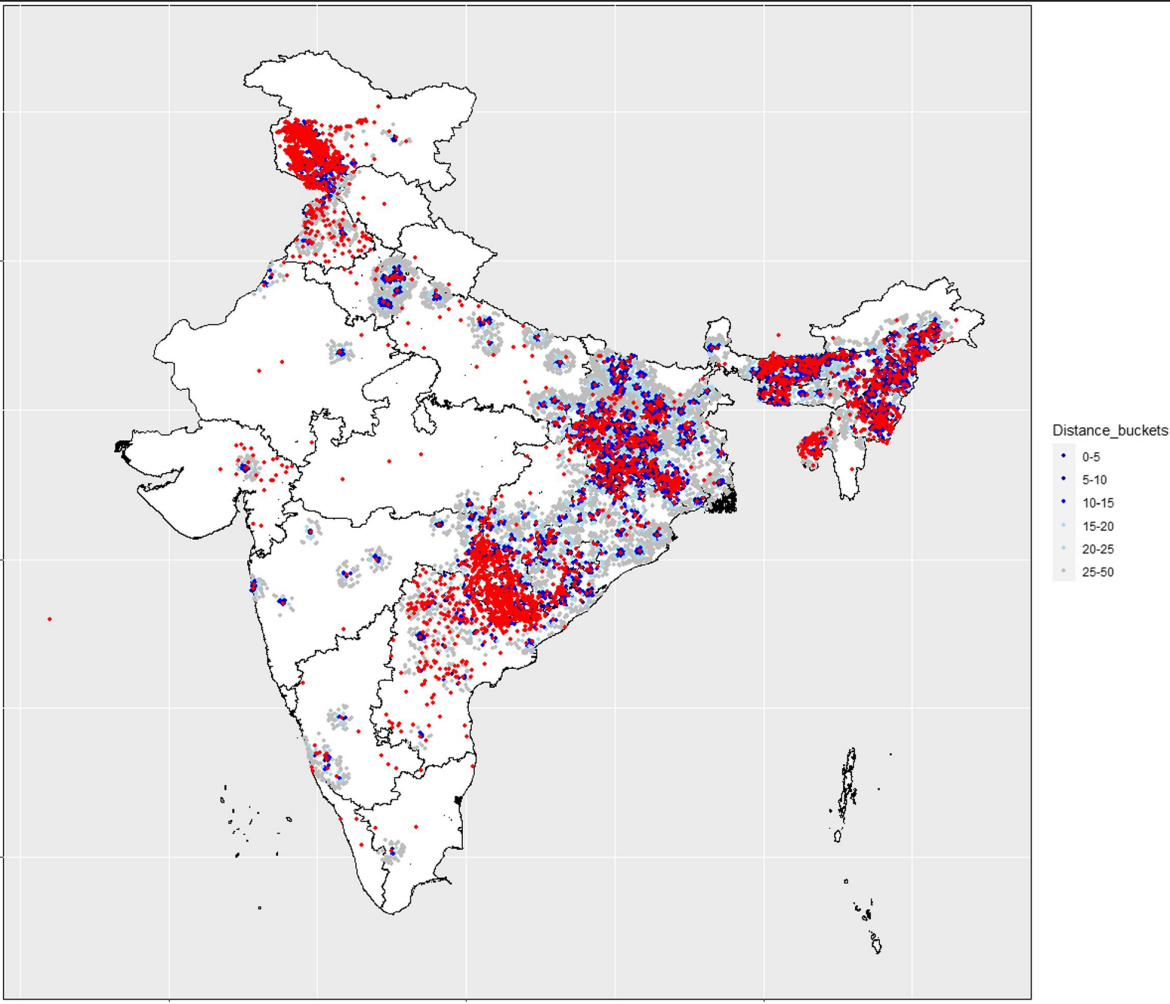
Map of India showing conflict events from Uppsala Conflict Data Program and residential clusters of sampled children in the National Family Health Survey, organized by increasing distance from site of conflict in kilometers. 1 km = 1.6 miles, so 50 km is about 31 miles

6245 violent events were recorded under UCDP, of which 4205 and 2040 events each were specified as exposure events for the NFHS 4 and NFHS-5 samples (Fig. 1, Additional file 1: Table S1, Fig. S2). Most of these were Maoist insurgencies in parts of central, western, eastern, and southern India (Additional file 1: Table S1). Violent events were clustered in the north, central and eastern regions (Fig. 1). Children across socioeconomic strata, religious groups, castes, and wealth quintiles were exposed to violence (Table 1) In the NFHS 4 (2015–16) sample, 13,085 of 28,518 clusters (villages or urban neighborhoods) were defined as ever conflict-exposed, of which 78,668 of 12,985 sampled children experienced conflicts (Table 1). In NFHS 5 and NFHS 4 samples, 20,638 and 41,393 children experienced conflicts in-utero or between 0 and 3 years respectively (Table 2). 8900 and 13,462 children (~ 11% each) experienced violence in-utero only, in NFHS 5 and 4 respectively; and 16,921 (~ 20%) and 22,817 (~ 18%) children experienced violence only during 0–3 years of age in NHFS 5 and 4 (Table 2). About 25% and 33% children in NFHS 5 and NFHS 4 respectively, experienced violence in both developmental periods (Table 2). Among the outcomes, in NFHS 5, mean and inter quartile range of HAZ, WAZ and WHZ were − 1.47 ( − 2.49,  − 0.35) SD,  − 1.42 ( − 2.24,  − 0.58) SD and  − 0.77 ( − 1.70, 0.12) SD respectively. Overall missingness in the outcomes in the sample ranged between 9.5 and 13.4%, with a higher missingness pattern in the exposed population, possibly since our design necessitated the date of outcome measurement in the survey to specify exposure (Table 2). Among the covariates, caste and mother’s height had under < 10% missingness (Table 1).

**Table 1:** Descriptive characteristics of sampled children in National Family Health Surveys (NFHS), India (2019–21 and 2015–16), stratified by estimated area level violence exposure in different child developmental periods

(A) NFHS-5 (2019–21)				
Variables	Children exposed to violence in-utero or within 0–3 years			
	N	Y	% of children exposed to violence (%)	*p* value
N	25,876	56,906	69	
Mother's education (%)				< 0.001
Higher	3211 (12.4)	5786 (10.2)	64	
No education	6078 (23.5)	14,066 (24.7)	70	
Primary	3544 (13.7)	7559 (13.3)	68	
Secondary	13,043 (50.4)	29,495 (51.8)	69	
Mother's height in cm [mean (SD)]	150.94 (6.16)	151.35 (6.34)		< 0.001
Missing	715	1054		
Religion (%)				< 0.001
Christian	3435 (13.3)	8163 (14.3)	70	
Hindu	18,002 (69.6)	35,489 (62.4)	66	
Muslim	3615 (14.0)	11,035 (19.4)	75	
Other	824 (3.2)	2219 (3.9)	96	
Caste (%)				< 0.001
Forward caste	3795 (14.7)	8598 (15.1)	69	
Other backward caste	9246 (35.7)	17,408 (30.6)	65	
Schedule caste	5337 (20.6)	9220 (16.2)	63	
Schedule tribe	5943 (23.0)	16,848 (29.6)	74	
Other	250 (1.0)	302 (0.6)	55	
Missing	1305 (5.0)	4530 (7.9)		
Wealth Quintile (%)				< 0.001
Middle	4525 (17.5)	9278 (16.3)	67	
Poorer	6292 (24.3)	14,460 (25.4)	70	
Poorest	8188 (31.6)	20,834 (36.6)	72	
Richer	3591 (13.9)	7077 (12.4)	66	
Richest	3280 (12.7)	5257 (9.2)	62	
Type of residence = urban (%)	5569 (21.5)	9525 (16.7)		< 0.001
Number of years in current residence [mean (SD)]	16.47 (27.51)	15.73 (25.82)		< 0.001
Child's gender = Male (%)	13,522 (52.3)	29,121 (51.2)		0.004
Age of Child (%)				< 0.001
0	4957 (19.2)	3991 (7.0)	45	
1	6147 (23.8)	8520 (15.0)	58	
2	4983 (19.3)	12,396 (21.8)	71	
3	2979 (11.5)	11,829 (20.8)	80	
4	3888 (15.0)	14,541 (25.6)	79	
5	2922 (11.3)	5629 (9.9)	66	

Variables	Children exposed to violence in-utero			
	N	Y	% of children exposed to violence (%)	*p*
N	46,741	36,041	44	
Mother's education (%)				< 0.001
Higher	5562 (11.9)	3435 (9.5)	38	
No education	11,105 (23.8)	9039 (25.1)	45	
Primary	6384 (13.7)	4719 (13.1)	43	
Secondary	23,690 (50.7)	18,848 (52.3)	44	
Mother's height in cm [mean (SD)]	151.07 (6.15)	151.43 (6.45)		< 0.001
Missing	1185	584		
Religion (%)				< 0.001
Christian	5688 (12.2)	5910 (16.4)	51	
Hindu	32,253 (69.0)	21,238 (58.9)	40	
Muslim	7111 (15.2)	7539 (20.9)	51	
Other	1689 (3.6)	1354 (3.8)	44	
Caste (%)				< 0.001
Forward caste	6897 (14.8)	5496 (15.2)	44	
Other backward caste	16,693 (35.7)	9961 (27.6)	37	
Schedule caste	9333 (20.0)	5224 (14.5)	36	
Schedule tribe	10,694 (22.9)	12,097 (33.6)	53	
Other	418 (0.9)	134 (0.4)	24	
Missing	2706 (5.8)	3129 (8.7)		
Wealth Quintile (%)				< 0.001
Middle	8011 (17.1)	5792 (16.1)	42	
Poorer	11,592 (24.8)	9160 (25.4)	44	
Poorest	15,063 (32.2)	13,959 (38.7)	48	
Richer	6323 (13.5)	4345 (12.1)	41	
Richest	5752 (12.3)	2785 (7.7)	33	
Type of residence = urban (%)	9581 (20.5)	5513 (15.3)		< 0.001
Number of years in current residence [mean (SD)]	15.87 (26.13)	16.09 (26.66)		0.238
Child's gender = male (%)	24,310 (52.0)	18,333 (50.9)		0.001
Age of child (%)				< 0.001
0	5106 (10.9)	3842 (10.7)	43	
1	8391 (18.0)	6276 (17.4)	43	
2	9022 (19.3)	8357 (23.2)	48	
3	7510 (16.1)	7298 (20.2)	49	
4	9840 (21.1)	8589 (23.8)	47	
5	6872 (14.7)	1679 (4.7)	20	

Variables	Children exposed to violence between 0 and 3 years			
	N	Y	% of children exposed to violence (%)	*p*
N	34,833	47,949	58	
Mother's education (%)				< 0.001
Higher	4237 (12.2)	4760 (9.9)	53	
No education	8010 (23.0)	12,134 (25.3)	60	
Primary	4729 (13.6)	6374 (13.3)	57	
Secondary	17,857 (51.3)	24,681 (51.5)	58	
Mother's height in cm [mean (SD)]	150.95 (6.19)	151.43 (6.35)		< 0.001
Missing	929	840		
Religion (%)				< 0.001
Christian	4812 (13.8)	6786 (14.2)	59	
Hindu	23,718 (68.1)	29,773 (62.1)	56	
Muslim	5166 (14.8)	9484 (19.8)	65	
Other	1137 (3.3)	1906 (4.0)	63	
Caste (%)				< 0.001
Forward caste	4994 (14.3)	7399 (15.4)	60	
Other backward caste	12,175 (35.0)	14,479 (30.2)	54	
Schedule caste	6878 (19.7)	7679 (16.0)	53	
Schedule tribe	8516 (24.4)	14,275 (29.8)	63	
Other	304 (0.9%)	248 (0.6)	45	
Missing	1966 (5.6)	3869 (8.0)	51	
Wealth Quintile (%)				< 0.001
Middle	6038 (17.3)	7765 (16.2)	56	
Poorer	8608 (24.7)	12,144 (25.3)	59	
Poorest	11,288 (32.4)	17,734 (37.0)	61	
Richer	4738 (13.6)	5930 (12.4)	56	
Richest	4161 (11.9)	4376 (9.1)	51	
Type of residence = urban (%)	7127 (20.5)	7967 (16.6)		< 0.001
Number of years in current residence [mean (SD)]	16.44 (27.72)	15.61 (25.33)		< 0.001
Child's gender = male (%)	18,014 (51.7)	24,629 (51.4)		0.323
Age of child (%)				< 0.001
0	8057 (23.1)	891 (1.9)	1	
1	8480 (24.3)	6187 (12.9)	42	
2	6742 (19.4)	10,637 (22.2)	61	
3	3913 (11.2)	10,895 (22.7)	74	
4	4664 (13.4)	13,765 (28.7)	75	
5	2977 (8.5)	5574 (11.6)	65	

(B) NFHS-4 (2015–16)				
Variables	Children exposed to violence in-utero or within 0–3 years			
	N	Y	% children exposed to violence (%)	*p*
N	46,510	78,668	63	
Mother's education (%)				< 0.001
No education	14,538 (31.3)	26,205 (33.3)	64	
Primary	6686 (14.4)	11,786 (15.0)	64	
Secondary	20,975 (45.1)	35,171 (44.7)	63	
Higher	4311 (9.3)	5506 (7.0)	56	
Mother's height [mean (SD)]	1608.05 (904.90)	1561.10 (658.29)		< 0.001
Missing	333	388		
Religion (%)				< 0.001
Christian	2577 (5.5)	11,719 (14.9)	82	
Hindu	33,095 (71.2)	48,280 (61.4)	59	
Muslim	8161 (17.5)	15,470 (19.7)	65	
Other	2677 (5.8)	3199 (4.1)	54	
Caste (%)				< 0.001
Schedule caste	9514 (21.2)	11,693 (16.1)	55	
Schedule tribe	7274 (16.2)	22,204 (30.6)	75	
Other backward caste	18,576 (41.3)	25,943 (35.8)	58	
Forward caste	9161 (20.4)	12,324 (17.0)	57	
Don't know	419 (0.9)	369 (0.5)	47	
Missing	1606	6095		
Wealth Quintile (%)				< 0.001
Poorest	12,376 (26.6)	25,526 (32.4)	67	
Poorer	10,522 (22.6)	21,555 (27.4)	67	
Middle	9194 (19.8)	14,998 (19.1)	62	
Richer	7645 (16.4)	9970 (12.7)	57	
Richest	6773 (14.6)	6619 (8.4)	49	
Type of residence = rural (%)	34,045 (73.2)	63,921 (81.3)		< 0.001
Number of Years in Current residence P[mean (SD)]	16.24 (27.46)	16.41 (26.52)		0.284
Child's sex = female (%)	21,969 (47.2)	38,159 (48.5)		< 0.001
Age of child (%)				< 0.001
0	8456 (18.2)	5039 (6.4)	37	
1	11,162 (24.0)	11,677 (14.8)	51	
2	9326 (20.1)	17,148 (21.8)	65	
3	6661 (14.3)	16,301 (20.7)	71	
4	6710 (14.4)	20,403 (25.9)	75	
5	4195 (9.0)	8100 (10.3)	66	

Variables	Children exposed to violence in-utero			
	N	Y	% children exposed to violence (%)	*p*
N	70,323	54,855	44	
Mother's education (%)				< 0.001
No education	22,343 (31.8)	18,400 (33.5)	45	
Primary	10,562 (15.0)	7910 (14.4)	43	
Secondary	31,298 (44.5)	24,848 (45.3)	44	
Higher	6120 (8.7)	3697 (6.7)	38	
Mother's height [mean (SD)]	1592.84 (837.02)	1560.20 (646.48)		< 0.001
Missing	452	269		
Religion (%)				< 0.001
Christian	5757 (8.2)	8539 (15.6)	60	
Hindu	48,647 (69.2)	32,728 (59.7)	40	
Muslim	12,238 (17.4)	11,393 (20.8)	48	
Other	3681 (5.2)	2195 (4.0)	37	
Caste (%)				< 0.001
Schedule caste	13,840 (20.5)	7367 (14.8)	35	
Schedule tribe	12,657 (18.7)	16,821 (33.8)	57	
Other backward caste	27,856 (41.2)	16,663 (33.4)	37	
Forward caste	12,792 (18.9)	8693 (17.5)	40	
Don't know	517 (0.8)	271 (0.5)	34	
Missing	2661	5040		
Wealth Quintile (%)				< 0.001
Poorest	19,389 (27.6)	18,513 (33.7)	49	
Poorer	16,527 (23.5)	15,550 (28.3)	48	
Middle	13,932 (19.8)	10,260 (18.7)	42	
Richer	11,100 (15.8)	6515 (11.9)	37	
Richest	9375 (13.3)	4017 (7.3)	30	
Type of residence = rural (%)	52,832 (75.1)	45,134 (82.3)		< 0.001
Number of years in current residence [mean (SD)]	16.55 (27.32)	16.08 (26.30)		0.002
Child's gender = female (%)	33,435 (47.5)	26,693 (48.7)		< 0.001
Age of child (%)				< 0.001
0	8583 (12.2)	4912 (9.0)	36	
1	12,859 (18.3)	9980 (18.2)	44	
2	14,011 (19.9)	12,463 (22.7)	47	
3	13,186 (18.8)	9776 (17.8)	43	
4	14,567 (20.7)	12,546 (22.9)	46	
5	7117 (10.1)	5178 (9.4)	42	

Variables	Children exposed to violence between 0 and 3 years			
	N	Y	% children exposed to violence (%)	*p*
N	60,968	64,210	51	
Mother's education (%)				< 0.001
No education	18,993 (31.2)	21,750 (33.9)	53	
Primary	8849 (14.5)	9623 (15.0)	52	
Secondary	27,676 (45.4)	28,470 (44.3)	51	
Higher	5450 (8.9)	4367 (6.8)	44	
Mother's height [mean (SD)]	1600.15 (872.35)	1558.02 (633.61)		< 0.001
Missing	392	329		
Religion (%)				< 0.001
Christian	4297 (7.0)	9999 (15.6)	70	
Hindu	42,921 (70.4)	38,454 (59.9)	47	
Muslim	10,395 (17.0)	13,236 (20.6)	56	
Other	3355 (5.5)	2521 (3.9)	43	
Caste (%)				< 0.001
Schedule caste	11,916 (20.3)	9291 (15.8)	44	
Schedule tribe	10,981 (18.7)	18,497 (31.5)	63	
Other backward caste	23,917 (40.7)	20,602 (35.1)	46	
Forward caste	11,421 (19.4)	10,064 (17.1)	47	
Don't know	499 (0.8)	289 (0.5)	37	
Missing	2234	5467	37	
Wealth Quintile (%)				< 0.001
Poorest	17,049 (28.0)	20,853 (32.5)	55	
Poorer	14,236 (23.3)	17,841 (27.8)	56	
Middle	11,999 (19.7)	12,193 (19.0)	50	
Richer	9591 (15.7)	8024 (12.5)	46	
Richest	8093 (13.3)	5299 (8.3)	40	
Type of residence = rural (%)	45,590 (74.8)	52,376 (81.6)		< 0.001
Number of years in current residence [mean (SD)]	16.15 (27.42)	16.54 (26.35)		0.011
Child's sex = female (%)	28,991 (47.6)	31,137 (48.5)		0.001
Age of child (%)				< 0.001
0	12,554 (20.6)	941 (1.5)	7	
1	14,994 (24.6)	7845 (12.2)	34	
2	12,045 (19.8)	14,429 (22.5)	55	
3	7629 (12.5)	15,333 (23.9)	67	
4	8425 (13.8)	18,688 (29.1)	69	
5	5321 (8.7)	6974 (10.9)	57

**Table 2:** Unadjusted national numbers and percentage of exposure(s) to conflicts by development period and prevalence of outcomes in conflict exposed clusters in National Family Health Surveys (NFHS) of India (2019–21 and 2015–16)

(A) Exposure to conflicts in each developmental period in the analytic sample				
Violence category	NFHS 5 (2019–21)		NFHS 4 (2015–16)	
	Number of children	% of children (%)	Number of children	% of children (%)
Experienced violence: in-utero only	8957	10.8	13,462	10.8
Experienced violence: 0–3 years only	20,865	25.2	22,817	18.2
Experienced violence: both in-utero and 0–3 years	27,084	32.7	41,393	33.1
Did not experience violence in-utero or 0–3 years	25,876	31.3	47,506	38.0
Total	82,782	100.0	125,178	100.0

(B) Prevalence of outcomes in each developmental period in the analytic sample				
	Children exposed to violence in-utero or within 0–3 years			
Variable	NFHS 5		NFHS 4	
	N	Y	N	Y
N	25,876	56,906	46,510	78,668
HAZ	− 1.28 ( − 2.36, − 0.03)	− 1.47 ( − 2.48, − 0.31)	− 1.31 ( − 2.32, − 0.11)	− 1.60 ( − 2.55, − 0.50)
Missing	5745 (22.2%)	1927 (3.3%)	10,677	2588
WAZ	− 1.34 ( − 2.17, − 0.50)	− 1.43 ( − 2.26, − 0.58)	− 1.38 ( − 2.18, − 0.49)	− 1.49 ( − 2.30, − 0.63)
Missing	5662 (21.9%)	1803 (3.2%)	10,677	2588
WHZ	− 0.81 ( − 1.70, 0.01)	− 0.79 ( − 1.60, 0.02)	− 0.86 ( − 1.73, 0.05)	− 0.82 ( − 1.66, 0.05)
Missing	5450 (21.1%)	1208 (2.12%)	10,677	2588

	Children exposed to violence in-utero			
Variable	NFHS 5	NFHS 4		
	N	Y	N	Y
N	46,741	36,041	70,323	54,855
HAZ	− 1.44 ( − 2.43, − 0.32)	− 1.40 ( − 2.48, − 0.12)	− 1.49 ( − 2.46, − 0.35)	− 1.52 ( − 2.51, − 0.37)
Missing	6635	1037	11,474	1791
WAZ	− 1.42 ( − 2.23, − 0.59)	− 1.40 ( − 2.25, − 0.52)	− 1.47 ( − 2.25, − 0.62)	− 1.44 ( − 2.27, − 0.54)
Missing	6510	955	11,474	1791
WHZ	− 0.80 ( − 1.60, 0.02)	− 0.77 ( − 1.63, 0.15)	− 0.85 ( − 1.69, 0.00)	− 0.81 ( − 1.68, 0.10)
Missing	5968	690	11,474	1791

	Children exposed to violence between 0 and 3 years			
Variable	NFHS 5	NFHS 4		
	N	Y	N	Y
N	34,833	47,949	60,968	64,210
HAZ	− 1.25 ( − 2.36, 0.05)	− 1.52 ( − 2.50, − 0.41)	− 1.29 ( − 2.33, − 0.07)	− 1.66 ( − 2.59, − 0.62)
Missing	6157	1515	11,133	2132
WAZ	− 1.34 ( − 2.18, − 0.49)	− 1.45 ( − 2.26, − 0.61)	− 1.37 ( − 2.19, − 0.45)	− 1.52 ( − 2.31, − 0.68)
Missing	6019	1446	11,133	2132
WHZ	− 0.81 ( − 1.70, 0.13)	− 0.79 ( − 1.54, 0.12)	− 0.85 ( − 1.74, 0.09)	− 0.82 ( − 1.64, 0.02)
Missing	5766	892	11,133	2132

### Association of conflict exposures and child anthropometric outcomes binary exposures

**Fig. 2:**
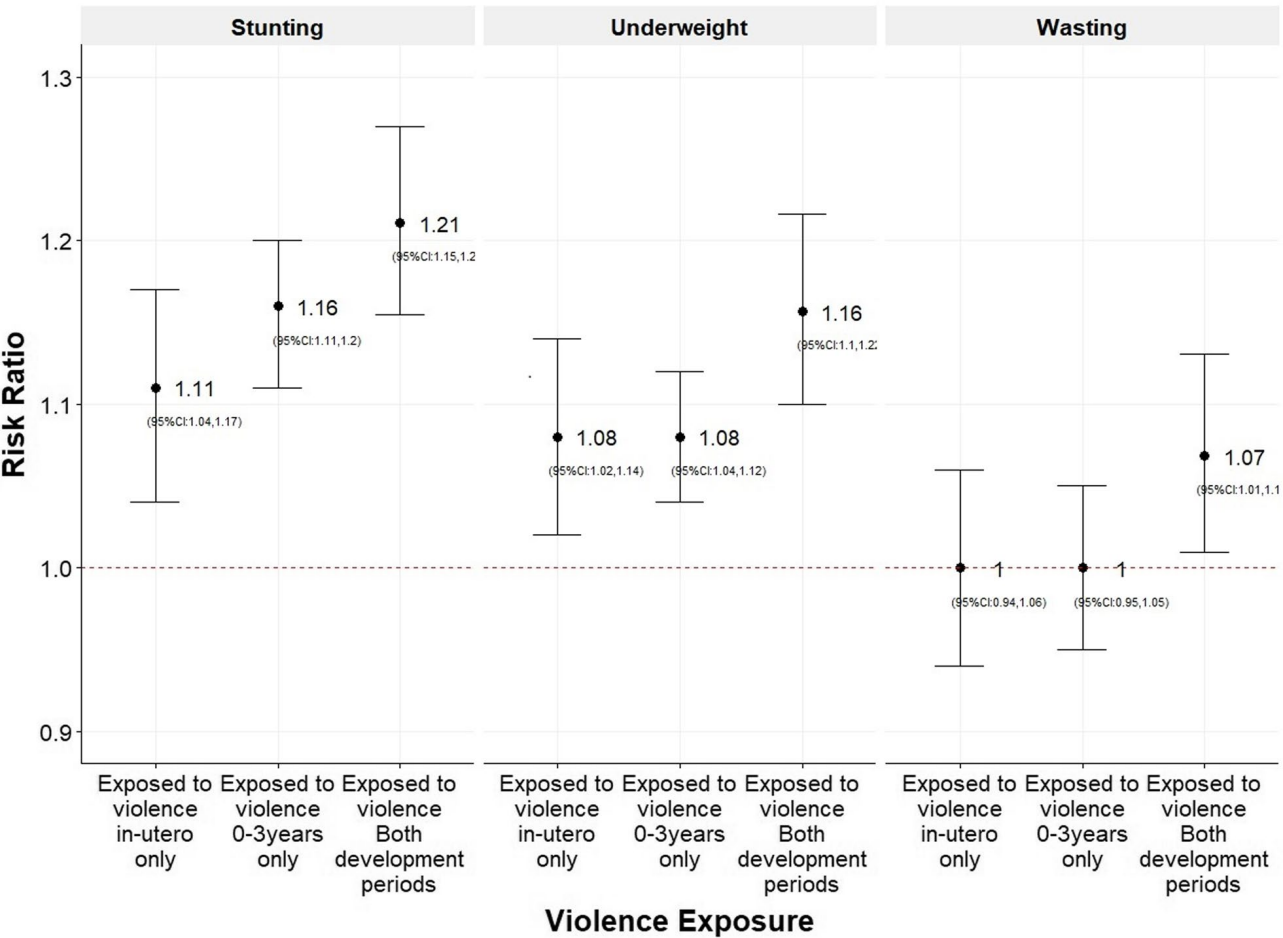
Predicted risk ratio of stunting, underweight and wasting for children sampled in NFHS 5 (2019–21) stratified by developmental period of violence exposure

In the NFHS 5 wave, in-utero exposure to violence was associated with risk ratios of 1.11 (95% CI 1.04–1.17) for stunting and 1.08 (95% CI 1.02–1.14) for underweight (Table 3A). Estimates were not statistically significant for wasting (Fig. 2) (Table 3). Exposure to violence between 0 and 3 years was associated with risk ratios of 1.16, (95% CI 1.11–1.20) for stunting, 1.08, (95% CI 1.04–1.12) for underweight, again with estimates not statistically significant for wasting (Fig. 2) (Table 3A). Children who experienced violence in-utero and in early childhood, had 1.21 (95% CI 1.15–1.27) times higher risks of stunting, 1.16 (95% CI 1.10–1.22) times higher risk of underweight, 1.07 (95% CI 1.01–1.13) times higher risk of wasting, compared to children who did not experience violence (Fig. 2) (Additional file 1: Table S2). Estimates for NFHS 4 were similar (Table 3B).

**Table 3:** Multivariate regression results for the association of violence on risks of child anthropometric outcomes and anthropometric z-scores nationally in National Family Health Survey of India (NFHS) (2019–21 and 2015–16)

(A) NFHS-5 (2019–21)									
Development period of violence exposure Binary exposure (Y or N)	Stunting			Underweight			Wasting		
	Risk ratio	95% CI	*p* value	Risk ratio	95% CI	*p* value	Risk ratio	95% CI	*p* value
In-utero exposure (in children <= 2 years)	1.11	1.04–1.17	0.00	1.08	1.02–1.14	0.01	1.00	0.94–1.06	0.89
0–3 years (in children <= 5 years)	1.16	1.11–1.2	0.00	1.08	1.04–1.12	0.00	1.00	0.95–1.05	0.95

Development period of violence exposure Binary exposure (Y or N)	HAZ			WAZ			WHZ		
	Estimate (mean difference)	95% CI	*p* value	Estimate (mean difference)	95% CI	*p* value	Estimate (mean difference)	95% CI	*p* value
In-utero exposure (in children <= 1 year)	− 0.11	− 0.19 to − 0.03	0.00	− 0.08	− 0.13 to − 0.03	0.00	0.05	− 0.02 to 0.12	0.16
In-utero exposure (in children <= 2 years)	− 0.07	− 0.12 to − 0.02	0.01	− 0.06	− 0.09 to − 0.02	0.00	0.02	− 0.03 to 0.06	0.48
0–3 years (in children <= 5 years)	− 0.12	− 0.15 to − 0.08	0.00	− 0.06	− 0.08 to − 0.03	0.00	− 0.06	− 0.09 to − 0.03	0.00
1st 1000 days (in children <= 5 years)	− 0.09	− 0.12 to − 0.06	0.00	− 0.06	− 0.08 to − 0.04	0.00	− 0.05	− 0.08 to − 0.03	0.00

(B) NFHS -4 (2015–16)									
Development period of violence exposure Binary exposure (Y or N)	Stunting			Underweight			Wasting		
	Risk ratio	95% CI	*p* value	Risk ratio	95% CI	*p* value	Risk ratio	95% CI	*p* value
In-utero exposure (in children <= 2 years)	1.12	1.06–1.17	0.00	1.12	1.06–1.18	0.00	1.06	1.00–1.12	0.04
0–3 years (in children <= 5 years)	1.19	1.15–1.23	0.00	1.13	1.09–1.17	0.00	1.10	1.06–1.15	0.00

Development period of violence exposure Binary exposure (Y or N)	HAZ			WAZ			WHZ		
	Estimate (mean difference)	95% CI	*p* value	Estimate (mean difference)	95% CI	*p* value	Estimate (mean difference)	95% CI	*p* value
In-utero exposure (in children <= 1 year)	− 0.11	− 0.17 to − 0.05	0.00	− 0.07	− 0.11 to − 0.03	0.00	0.02	− 0.03 to 0.08	0.44
In-utero exposure (in children <= 2 years)	− 0.07	− 0.11 to − 0.03	0.00	− 0.05	− 0.08 to − 0.03	0.00	− 0.01	− 0.04 to 0.03	0.71
0–3 years (in children <= 5 years)	− 0.13	− 0.16 to − 0.11	0.00	− 0.08	− 0.1 to − 0.06	0.00	− 0.07	− 0.09 to − 0.05	0.00
1st 1000 days (in children <= 5 years)	− 0.10	− 0.12 to − 0.07	0.00	− 0.06	− 0.07 to − 0.04	0.00	− 0.03	− 0.05 to − 0.01	0.00

For each developmental period of exposure (each row), the reference category is children who experienced no violence in that developmental period

### Chronicity and intensity of exposure

In both NFHS 5 and 4, incremental quartiles of exposure to violent events and deaths had increasing estimated risks of stunting and underweight until quartile 3, after which they decreased, thus indicating a U-shaped relationship (relative to children who did not experience any conflicts) (Table 4A and B) (Additional file 1: Fig. S2). For wasting, estimates were not statistically significant at *p *<= 0.05. In NFHS 5, for in-utero exposures, quartiles 1–4 of number of conflict events were associated with 1.06 (95% CI 0.99–1.14), 1.12 (95% CI − 1.02 to 1.24), 1.08 (95% CI 0.95–1.23) and 1.29 (95% CI 1.16–1.43) times higher risks of stunting, and 1.04 (95% CI 0.98–1.12), 1.13 (1.02, 1.25), 1.10 (95% CI 0.97–1.25), and 1.16 (95% CI 1.04–1.29) times higher risks of underweight, in children <= 2 years (Table 4A).

**Table 4:** Multivariate regression results for the association of quartiles of violence exposure on child anthropometric outcomes and anthropometric Z-scores nationally in Nation Family Health Surveys (2019–21 and 2015–16)

(A) NFHS- 5 (2019–21)									
Quartile of violence events exposure In-utero exposure (in children <= 2 years)	Stunting			Underweight			Wasting		
	Risk ratio	95% CI	*p* value	Risk ratio	95% CI	*p* value	Risk ratio	95% CI	*p* value
Quartile 1	1.06	0.99–1.14	0.08	1.04	0.98–1.12	0.22	1.01	0.94–1.09	0.83
Quartile 2	1.12	1.02–1.24	0.02	1.13	1.02–1.25	0.02	0.99	0.89–1.11	0.91
Quartile 3	1.08	0.95–1.23	0.23	1.10	0.97–1.25	0.15	1.01	0.87–1.16	0.93
Quartile 4	1.29	1.16–1.43	0.00	1.16	1.04–1.29	0.01	0.95	0.84–1.07	0.37

Quartile of violence events exposure 0–3 years (in children <= 5 years)	Stunting			Underweight			Wasting		
	Risk ratio	95% CI	*p* value	Risk ratio	95% CI	*p* value	Risk ratio	95% CI	*p* value
Quartile 1	1.08	1.04–1.13	0.00	1.03	0.99–1.08	0.00	1.03	0.98–1.09	0.21
Quartile 2	1.07	1.02–1.12	0.00	1.07	1.02–1.12	0.10	1.06	1.00–1.12	0.81
Quartile 3	1.11	1.04–1.18	0.00	1.12	1.05–1.2	0.36	1.10	1.01–1.19	0.60
Quartile 4	1.10	0.99–1.21	0.22	1.26	1.14–1.39	0.25	1.23	1.10–1.39	0.00

(B) NFHS -4 (2015–16)									
Quartile of violence events exposure 0–3 years (in children <= 5 years)	Stunting			Underweight			Wasting		
	Risk ratio	95% CI	*p* value	Risk ratio	95% CI	*p* value	Risk ratio	95% CI	*p* value
Quartile 1	1.05	1.01–1.09	0.01	1.05	1.01–1.09	0.02	1.03	0.99–1.08	0.15
Quartile 2	1.01	0.97–1.06	0.55	1.08	1.04–1.13	0.00	1.10	1.05–1.16	0.00
Quartile 3	0.99	0.94–1.04	0.62	1.16	1.09–1.22	0.00	1.32	1.24–1.4	0.00
Quartile 4	1.07	0.98–1.18	0.14	1.19	1.08–1.31	0.00	1.36	1.22–1.52	0.00

Quartile of violence events exposure In-utero exposure (in children <= 2 years)	Stunting			Underweight			Wasting		
	Risk ratio	95% CI	*p* value	Risk ratio	95% CI	*p* value	Risk ratio	95% CI	*p* value
Quartile 1	1.11	1.05–1.18	0.00	1.09	1.02–1.16	0.01	1.02	0.95–1.09	0.65
Quartile 2	1.01	0.93–1.09	0.82	1.09	1.01–1.18	0.03	1.08	0.99–1.18	0.07
Quartile 3	1.11	1–1.23	0.05	1.08	0.97–1.2	0.16	1.05	0.94–1.18	0.40
Quartile 4	1.27	1.16–1.4	0.00	1.32	1.2–1.45	0.00	1.18	1.07–1.31	0.00

Estimates and CIs are on the risk ratio scale

For each quartile of violence exposure (each row), the reference category is children who experienced no violence (i.e., their Risk Ratio was 1)

### Effect measure modification

Exposure to violence was associated with poor anthropometric outcomes in all children, indicating no statistically significant effect modification patterns by children’s socioeconomic characteristics or minority social group identity. However, minority and poor children, who had higher prevalence of stunting, underweight and wasting in the absence of violence, did incrementally worse under violence exposure (Additional file 1: Fig. S3). Female children who had an advantage over males in the absence of conflicts, reduced this benefit under violence exposure (Additional file 1: Fig. S3). Christian children who had better outcomes than Hindus, also lost this gain under violence exposure (Additional file 1: Fig. S3). Among different types of conflicts, police violence, insurgencies in the north-east, Hindu Muslim riots, and Maoist violence had the highest negative associations with anthropometric outcomes, although confidence intervals were overlapping (Additional file 1: Fig. S4). In Kashmir, children exposed to violence had lower prevalence of stunting.

### Secondary outcomes

Children exposed to violence in-utero had 1.07 (95% CI 1.04–1.10) times and 1.06 (95% CI 1.03–1.10) times higher risks of low birth weight in NFHS 4 and 5 respectively (Additional file 1: Table S3). After controlling for maternal anemia, children exposed to violence had higher risks of anemia (RR: 1.10 [95% CI 1.06–1.13]) (Additional file 1: Table S4). Mothers who were exposed to violence also had higher risks of anemia (Additional file 1: Table S4).

### Comparison of estimates from the NFHS 4 and NFHS 5 samples

Estimates for NFHS 4 were similar to NFHS 5, for stunting, underweight and wasting. For wasting, the low effect sizes in NFHS 4, dropped further in NFHS 5. On violence intensity and chronicity, estimates for NFHS 4 were marginally higher than NFHS 5.

### Sensitivity analyses

#### Sibling subsample

In 17,760 siblings born to 8333 mothers, with discordant conflict exposure, in NFHS 5, exposure to violence between 0 and 3 years was associated with 1.19 times (95% CI 1.10–1.29) higher risks of stunting, and correspondingly, an average decrease in HAZ of 0.24 SD (95% CI − 0.33 to − 0.16) (Additional file 1: Table S5). In NFHS 4, an average decrease in HAZ of 0.17 SD (95% CI − 0.24 to − 0.08) was observed in the sibling sub sample.

#### Confounding from COVID-19 for NFHS 5

Unadjusted prevalence of stunting, wasting and underweight from states which sampled most children pre lockdown and those which sampled children both pre and post COVID-19 lockdown were similar (Additional file 1: Table S11).For the 22 states where > 95% children were sampled pre Covid, exposure to violence in-utero was associated with 1.21 times (95% CI 1.12–1.31) higher risks of stunting and 1.11 (95% CI 1.02–1.19) times higher risk of underweight (Additional file 1: Table S6). Exposure to violence in early childhood was associated with 1.12 times (95% CI 1.06–1.18) higher risks of stunting and 1.09 times (95% CI 1.03–1.15) higher risk of underweight (Additional file 1: Table S6). These estimates were consistent with the larger samples in effect sizes, and more precise. Estimates for wasting were similar, and unlike the larger NFHS 5 sample, were statistically significant at *p* = 0.05 level.In 13 states where children were sampled both pre and post COVID lockdown, exposure to violence in-utero, was associated with 1.04 times (95% CI 0.91–1.08) higher risks of stunting and 1.04 (95% CI 0.96–1.13) times higher risk of underweight. Exposure to violence in early childhood was associated with 1.21 times (95% CI 1.15–1.28) higher risks of stunting and 1.06 (95% CI 1.02–1.12) times higher risk of underweight (Additional file 1: Table S7). While estimates were consistent with the larger samples, they were reduced in effect size, especially in the case of in-utero exposures to conflicts (Additional file 1: Table S7).

#### Distance from site of conflict

For children exposed to violence in-utero—5 years of age, for every 10% increase in distance from the site of conflict, anthropometric outcomes improved (Additional file 1: Table S8). For HAZ, the increase was 0.003 SD (95% CI 0.001–0.0005) (Additional file 1: Table S1.10). Additionally, to assess possible bias from the 50 km distance cut-off we used to define conflict exposed sites, we assessed estimates with other distance limits. Estimates increased progressively for 50, 75 and 100 km, before dropping at 200 km for HAZ (Additional file 1: Table S8). All results estimated with the 100 km cut-off are presented for comparison (Additional file 1: Table S12).

## Discussion

We investigated prolonged internal conflicts, characteristic of multiethnic, multireligious societies in many LMICs, as determinants of sub-optimal child growth in India. Our analysis, covering over a decade of conflicts and two recent waves of nationally representative child health data, found exposure to violence during two critical child developmental periods, namely in-utero and in early childhood, were associated with increments in stunting and underweight. For wasting, associations were only found for children exposed to violence in-utero and between 0 and 3 years, but not in either period alone. Our findings were robust in a sub-sample of siblings who were discordant in conflict exposures, indicating robustness to time-invariant household characteristics which may be correlated with the exposure and outcomes.

In fact, estimates in the sibling subsamples were marginally higher than the larger sample, similar to Duque et al.’s [59] findings in Columbia. We found the strongest associations for stunting for children exposed to violence between 0 and 3 years. Also, children exposed to conflicts in both developmental periods had higher risks of stunting, compared to children exposed in either period alone (Additional file 1: Table S2). This aligns with scholarship on the accrued harms of multiple negative exposures in life course epidemiology [38, 60]. However, we also found children who experienced more frequent and more intense conflicts (conflicts with more deaths), experienced a thresholding effect after quartiles 2 and 3 of events, respectively [61]. These findings are similar Krief et al.’s [7] results in Colombia, where exposure to intermittent conflicts were associated with stronger negative associations for both child anthropometry and utilization of prenatal care in municipalities, compared to exposure to continuous conflicts. The authors hypothesized this to be due to adaptative mechanisms developed in the case of chronic exposure to adversities [7, 9]. While we did not quantitatively examine this hypothesis in our analysis, such coping mechanisms could also apply in our case.

In contrast to studies focused on ubiquitous, national exposures to conflicts, in our study, longstanding internal conflicts only affected specific areas (Fig. 1). Given this internal geographical patterning of political violence, we followed Wagner et al.’s [43, 44] identification strategy in African countries to restrict our analysis to conflict exposed areas, while exploiting temporal variations in children’s indirect conflict exposure. Thus, our analysis was based on a relatively conservative design, where the counterfactual group also lived in violence exposed areas but did not experience violence in specific developmental periods under consideration. Yet, our estimates were somewhat higher than two other studies focused on chronic conflicts, both from Columbia. In the first, exposure to massacres in the second and third trimester of pregnancy, and between 0 and 3 years, were associated with 0.04, 0.03 and 0.09 SD decline in HAZ, respectively [59]. A second study, found exposure to higher intensity conflicts in-utero caused 0.06 SD lower HAZ and WAZ each, and 0.03 SD lower WHZ; and similar exposures in year 1, 2 and 3 caused 0.02, 0.07 and 0.02 SD lower HAZ respectively; 0.03, 0.04 and 0.03 SD lower WAZ; and 0.02, 0.00 and 0.05 lower SD WHZ, compared to lower intensity exposures [7].

While the violent events in our analysis were of lower intensity and scale, our estimates were comparable to some studies from war settings that have studied sub-optimal growth in its association with conflict exposure, and lower than others. In Iraq, district level violence exposure was associated with a 0.006 and 0.019 SD drop in HAZ in 2–5-year-old children [28]. However, other high intensity conflicts, from Rwanda, Burundi, and Côte d'Ivoire, showed effect sizes higher than ours, ranging from 0.2 to 0.5 SD lower HAZ in war exposed children (Additional file 1: Table S13) [24, 27, 34]. As hypothesized by Duque et al., [59] large scale wars concentrated in specific areas, could be causing more devastating disruptions in food supply and health services, compared to long-simmering conflicts over larger areas. Importantly, while our findings were focused on India, they have ramifications for other LMICs which have a history of protracted conflicts. Importantly, while many attributes of their deleterious effects on child health are likely to be similar across contexts, the types and characteristics of conflicts including their chronicity and intensity, resilience of health and nutritional support systems, are likely to also cause variations [1, 24, 34].

We studied conflict exposure in two important sensitive periods for child development [22]. Similar to Duque et al.’s [59] findings in Columbia, we found strongest negative associations in child growth for violence exposure in early childhood (0–3 years). Early childhood environment has been associated strongly with growth, with many studies examining the role of optimal nutrition environments, parental support and school environments as specific environmental stressors influencing neurodevelopmental pathways [22, 62, 63]. For in-utero exposures, child height is has been associated with maternal nutritional deprivation later in the pregnancy, and with nutritional disruptions in early childhood [33, 38, 64].

In addition to indicators of suboptimal growth, we found higher risks of childhood anemia in conflict exposed children, a secondary outcome in our analysis. This finding supports scholarship that has reasoned how extended conflicts may interrupt local food supply systems, which in turn could be leading to population level nutritional deficiencies in children [28]. While we were unable to quantitatively examine these other hypotheses, studies have also posited other pathways for how area level conflict exposure may be associated with deleterious child growth. For example, it has been proposed that conflicts could adversely affect nutrition enabling environments at home and in school, which may impact child growth [65]. Moreover, maternal stress during conflicts has been associated with lower birth weight, small gestational age and preterm births, which have in turn been associated with poor growth [22]. In our analysis, we also found higher risks for low-birth weight for in-utero violence exposures, another secondary outcome in our analysis. This is aligned with evidence on how perinatal nutritional disruptions and neurobiological maternal stress pathways may be associated with poor birth outcomes, which may lead to disadvantageous child health and developmental outcomes [22].

We did not find evidence of effect modification by child’s social group or sex, in the risks of sub-optimal growth under violence exposure (Additional file 1: Fig. S3). This may suggest that area level violence has equally negative associations for growth outcomes for all children, irrespective of their social group identity. However, it is important to underscore here that the UCDP data we used to measure exposure to conflicts excluded conflicts which targeted minority children, namely caste and communal violent events, due to a key inclusion criteria to only capture conflict events which caused  >= 25 deaths annually [12, 53]. Thus, alternate data sources and experimental designs may be needed for a focused study on how such conflicts in India which may disproportionately affect minority children, influence child health. Moreover, despite the limitations in our analysis, we did find that children from minority religions and castes, who already had a higher prevalence of stunting and underweight, fell still further behind their more advantaged counterparts, when they were exposed to conflicts (Additional file 1: Fig. S3). Thus, our findings also lend some evidence to the possible role of extended conflicts in aggravating nutritionally disadvantageous positions of minority children [13]. Thus, intervening on conflicts could be important to mitigate population level child growth disparities in India [66].

Additionally, we also found that the advantage female children otherwise had over males in anthropometric z-scores, diminished under conflict exposure (Additional file 1: Fig. S3). This may indicate that in conflict situations, under scarcity of resources, families allocate more resources towards boys. This hypothesis is supported by many studies on preference of the male child in the Indian subcontinent [67].

While we did not identify effect modification patterns by types of violence, we found longstanding internal political struggles including insurgencies in the North–east, Naxal-Maoist violence in central India and Hindu-Muslim sectarian violence in different parts of the country had similarly deleterious effects on sub-optimal growth (Additional file 1: Fig. S4). Given the different socio-political histories of these conflicts, their affected populations, alternate study designs and data sources may be required to study how different types of extended violence influence child growth variably in India. For instance, in an apparently contradictory finding, we found children exposed to violence seemingly had better outcomes in J& K. This was possibly because 80% sampled children in the union territory were exposed to violence, compared to 32% nationally, thus indicating the need for a more suitable counterfactual population, thus suggesting alternate identification strategies might be needed to quantify the extent of the impact of conflict exposure in Kashmir in detail (Additional file 1: Fig. S4). This could also be partially attributed to the higher resiliency of children chronically exposed to violence, [68, 69] as well as high protein dietary patterns and the improved health and sanitation indicators in the region [70]. However, we did find that children who experienced higher quartiles of number of events and deaths did have poorer outcomes in J&K, indicating higher intensity and frequency of violence exposures had incrementally deleterious outcomes. Thus, in chronically violence exposed regions, variations in violence intensity may be a more suitable exposure measure than specifications around binary exposure to violence (exposed to violence vs not).

Broadening the definition of conflict exposed clusters to <= 75 and <= 100 km increased our estimated effect sizes. This could be due to the concentration of violent events in rural and hilly areas, and the large size of districts (average diameters of 79 km) [40], which comprise administrative and police jurisdiction units in India [71]. Information on violent events also likely spreads through mass and social media, increasing the likelihood of spillover effects beyond neighborhoods [5]. Thus, violence exposed areas could have been misclassified as unexposed, which could have underestimated our estimates.

We did not find statistically significant associations of conflict events with wasting or child WHZ. While stunting is an indicator of chronic undernutrition and wasting points to more recent food deprivation or illnesses, underweight captures both [54]. Since we observed stronger, more precise associations for stunting, our findings could be reflective of longer-term disruptions of development potential [2, 65].

We found higher effect sizes for children sampled in NFHS 4, compared to NFHS 5, possibly since more violent incidents were included in NFHS 4 in our study design (4205 compared to 2040) (Additional file 1: Table S1, Fig. S1). Finally, how could COVID-19 have confounded our results for NFHS 5? First, our estimates for NFHS-5 and the pre-COVID NFHS-4 wave were largely consistent. Second, we found no differences in unadjusted prevalence of stunting, wasting and underweight from states which sampled a critical mass of children both pre and post COVID-19 lockdown (Additional file 1: Table S11). Third, in the restricted sample from 22 states which sampled >92% children pre-COVID-19 lockdown, estimates were comparable to NFHS 4 (Additional file 1: Table S6). Furthermore, in the subsample of 13 states where children were sampled in both pre/post Covid lockdown phases, estimates were somewhat reduced in effect size especially for in-utero exposures, but directionally similar to both NFHS 5 and 4 samples (Additional file 1: Table S7). This could be due to reduced violence events during the COVID-19 lockdown (Additional file 1: Fig. S1) such that the probability of a child <2 years (or < 1 year) sampled in the post lockdown phase to be exposed to violence under our study design was much reduced.

Our study had several limitations. The UCDP data we used to measure children’s exposure to conflicts excluded caste and communal violent events which did not cause  >= 25 deaths annually. There could be measurement error in children’s age, given poor birth registration, and possible recall bias of interviewed mothers. Our data were cross-sectional, with a possibility of unmeasured confounding. NFHS geospatial coordinates were displaced by up to 5 km in 99% of rural clusters, leaving room for misclassification of conflict exposed clusters [46]. Since hemoglobin and anthropometric measurements were collected cross-sectionally, we could not do a mediation analysis to investigate mechanisms. Despite sensitivity tests, confounding from the COVID-19 lockdown is possible for the NFHS 5 sample. We treated all pregnancies as full term in estimating in-utero exposures and did not account for preconception exposures, possibly biasing our estimates downwards. Since the date of measurement of anthropometry was necessary to temporarily specify exposure, there was a higher missingness pattern among the unexposed children. However, since this was still less than 13%, we did not impute any data.

Despite these limitations, our study had many strengths. We investigated protracted internal conflicts over two repeated cross-sections of nationally representative data in India and identified its deleterious associations with short- and long-term child growth. Our estimates were robust to multiple sensitivity tests, including in sibling subsamples. If anything, we underestimated effects by not capturing all types of protracted violence.

## Conclusion

In-utero and early childhood indirect exposure to protracted conflicts were associated with increased stunting and underweight in India. Given the continued exposures of such historically and contextually rooted internal conflicts in many LMICs, chronic violence exposures should be targeted in public health policies as social and political determinant of child health.

## Supplementary Information

**Additional file 1: Figure S1.** Number of high intensity conflict events and deaths by year from Uppsala Conflict Data Program’s India data. **Figure S2 (A):** Predicted prevalence of stunting and underweight nationally for quartiles of violence exposure for children 0-3 years, for National Family Health Survey-5 (2019-21). The red line indicates the predicted prevalence of underweight for children who were not exposed to conflicts and **Figure S2 (B):** Predicted prevalence of stunting and underweight nationally for quartiles of violence exposure for children 0-3 years, for National Family Health Survey-4 (2015-16). The red line indicates the predicted prevalence of underweight for children who were not exposed to conflicts. **Figure S3.** Effect measure modification by different socioeconomic characteristics in the association of violence exposure between 0 and 3 years of age and child Height-For-Age-Z-scores, National Family Health Survey. **Figure S4.** Effect measure modification by types of chronic violence, in the association of exposure to conflicts in 0-3 years, and predicted probabilities of stunting in children <=5 years, as an example. Here the red line indicates predicted prevalence of stunting under no violence exposure, which is the reference group. **Figure S5:** Association between violence exposure between 0-3 years and HAZ by type of violence and distance from conflict. **Figure S6:** Trends in month/year of height and weight measurement of sampled children in NFHS 5 (2019-21). **Figure S7: Flow chart explaining study design with three illustrative children in sample.** Here the flashy sparks indicate violent events. Child 1 experiences violence both in-utero and during 0-3 years, child 2 is born in January 17, before any violent events, so is treated as unexposed for in-utero exposures, and exposed for early childhood exposures. Child 3 who is conceived after violent events, is treated as unexposed for both developmental periods. This highlights two limitations in our design which possibly underestimated exposure to violence- 1) all pregnancies were treated as full term, 2) pre-conception exposures were not accounted for. **Table S1.** Types of high intensity, protracted violent events by year from Uppsala Conflict data program’s data for India, 2009-2020. **Table S2.** Multivariate regression results for the association of violence on risks of child anthropometric outcomes comparing children exposed in single and multiple developmental periods in National Family Health Survey of India-5 (NFHS) (2019-21). **Table S3.** Multivariate regression results for association between in-utero violence exposure and birth weight in children < =5 years in National Family Health Surveys (NFHS) 2015-16 and 2019-21. **Table S4.** Multivariate regression results for association between violence exposure on maternal and childhood anemia using National Family Health Surveys (NFHS) 2015-16 and 2019-21. **Table S5.** Multivariate regression results for association between in-utero violence exposure and anthropometric outcomes in children < 2 years, who had at least one sibling who did not experience violence. Data: National Family Health Surveys (NFHS) 2015-16 and 2019-21. **Table S6.** Multivariate regression results for the association of violence exposure on child anthropometric Z- scores nationally in states which were almost entirely (92%) sampled pre COVID National Family Health Survey (2019-21). **Table S7.** Multivariate regression results for the association of violence exposure on child anthropometric Z- scores nationally in states which sampled both pre and post COVID, National Family Health Survey (2019-21). **Table S8**. Estimating influence of distance thresholds from site of conflict on association of conflict exposure and child anthropometry (with HAZ as an example). **Table S9. **National number and percentage of children sampled by month of their height and weight measurement in National Family Health Survey (2019-21). **Table S10.** Distribution of sampled children and the percentage of children sampled pre-COVID by States. Here, Pre-covid cut-off has been defined as pre 3/25/2020, based on distributions of sampled children in Table 1 and Figure 1. **Table S11.** Unadjusted prevalence of anthropometric outcomes in states sampled almost entirely in the pre-COVID lockdown phase, and states which sampled children both pre and post COVID lockdown phases. **Table S12**. Multivariate regression results for the association of violence exposure on child anthropometric Z- scores nationally (distance cut-off: 100 km) in National Family Health Surveys (2015-16). **Table S13.** Comparison of effect sizes of HAZ in the present study with other studies.

## Data Availability

All data and materials are publicly available.
