# Supplementary material for: Area level indirect exposure to extended conflicts and early childhood anthropometric outcomes in India: a repeat cross-sectional analysis
Source: Confl Health. 2023 May 7;17:23. doi: 10.1186/s13031-023-00519-8 (PMC10164367; doi:10.1186/s13031-023-00519-8)
Supplement: Supplementary file 1 — Additional file 1: Figure S1. Number of high intensity conflict events and deaths by year from Uppsala Conflict Data Program’s India data. Figure S2 (A): Predicted prevalence of stunting and underweight nationally for quartiles of violence exposure for children 0-3 years, for National Family Health Survey-5 (2019-21). The red line indicates the predicted prevalence of underweight for children who were not exposed to conflicts and Figure S2 (B): Predicted prevalence of stunting and underweight nationally for quartiles of violence exposure for children 0-3 years, for National Family Health Survey-4 (2015-16). The red line indicates the predicted prevalence of underweight for children who were not exposed to conflicts. Figure S3. Effect measure modification by different socioeconomic characteristics in the association of violence exposure between 0 and 3 years of age and child Height-For-Age-Z-scores, National Family Health Survey. Figure S4. Effect measure modification by types of chronic violence, in the association of exposure to conflicts in 0-3 years, and predicted probabilities of stunting in children <=5 years, as an example. Here the red line indicates predicted prevalence of stunting under no violence exposure, which is the reference group. Figure S5: Association between violence exposure between 0-3 years and HAZ by type of violence and distance from conflict. Figure S6: Trends in month/year of height and weight measurement of sampled children in NFHS 5 (2019-21). Figure S7: Flow chart explaining study design with three illustrative children in sample. Here the flashy sparks indicate violent events. Child 1 experiences violence both in-utero and during 0-3 years, child 2 is born in January 17, before any violent events, so is treated as unexposed for in-utero exposures, and exposed for early childhood exposures. Child 3 who is conceived after violent events, is treated as unexposed for both developmental periods. This highlights two limitations in our design [file 13031_2023_519_MOESM1_ESM.docx]

**Supplementary Tables and Figures**

**Table S1:** Types of high intensity, protracted violent events by year from Uppsala Conflict data program’s data for India, 2009-2020

| **Type of Violence events** | **2009** | **2010** | **2011** | **2012** | **2013** | **2014** | **2015** | **2016** | **2017** | **2018** | **2019** | **2020** | **% of Total** |
| --- | --- | --- | --- | --- | --- | --- | --- | --- | --- | --- | --- | --- | --- |
| Maoist/Naxal Insurgency | 354 | 422 | 252 | 215 | 160 | 155 | 149 | 222 | 170 | 204 | 167 | 132 | 45.2% |
| India: Kashmir | 233 | 221 | 112 | 83 | 73 | 103 | 85 | 117 | 146 | 179 | 128 | 152 | 29.6% |
| North-East insurgency | 251 | 122 | 40 | 125 | 69 | 117 | 62 | 54 | 35 | 31 | 12 | 10 | 18.5% |
| Border conflicts |  | 7 | 2 | 5 | 12 | 23 | 24 | 26 | 40 | 60 | 46 | 46 | 4.7% |
| Hindu-Muslim riots |  | 2 |  |  | 19 |  |  |  | 7 |  |  | 41 | 1.1% |
| Government Perpetrated | 4 | 4 | 1 |  | 11 | 2 | 1 |  | 2 | 4 | 1 | 8 | 0.6% |
| Terrorism |  | 2 | 1 |  | 2 |  |  |  |  |  |  |  | 0.2% |
| Sikh insurgency | 1 |  |  |  |  |  |  |  |  |  |  |  | 0.0% |
| **Grand Total** | **843** | **780** | **408** | **428** | **346** | **400** | **321** | **419** | **400** | **478** | **354** | **389** | **100%** |
|  |  |  |  |  |  |  |  |  |  |  |  |  |  |


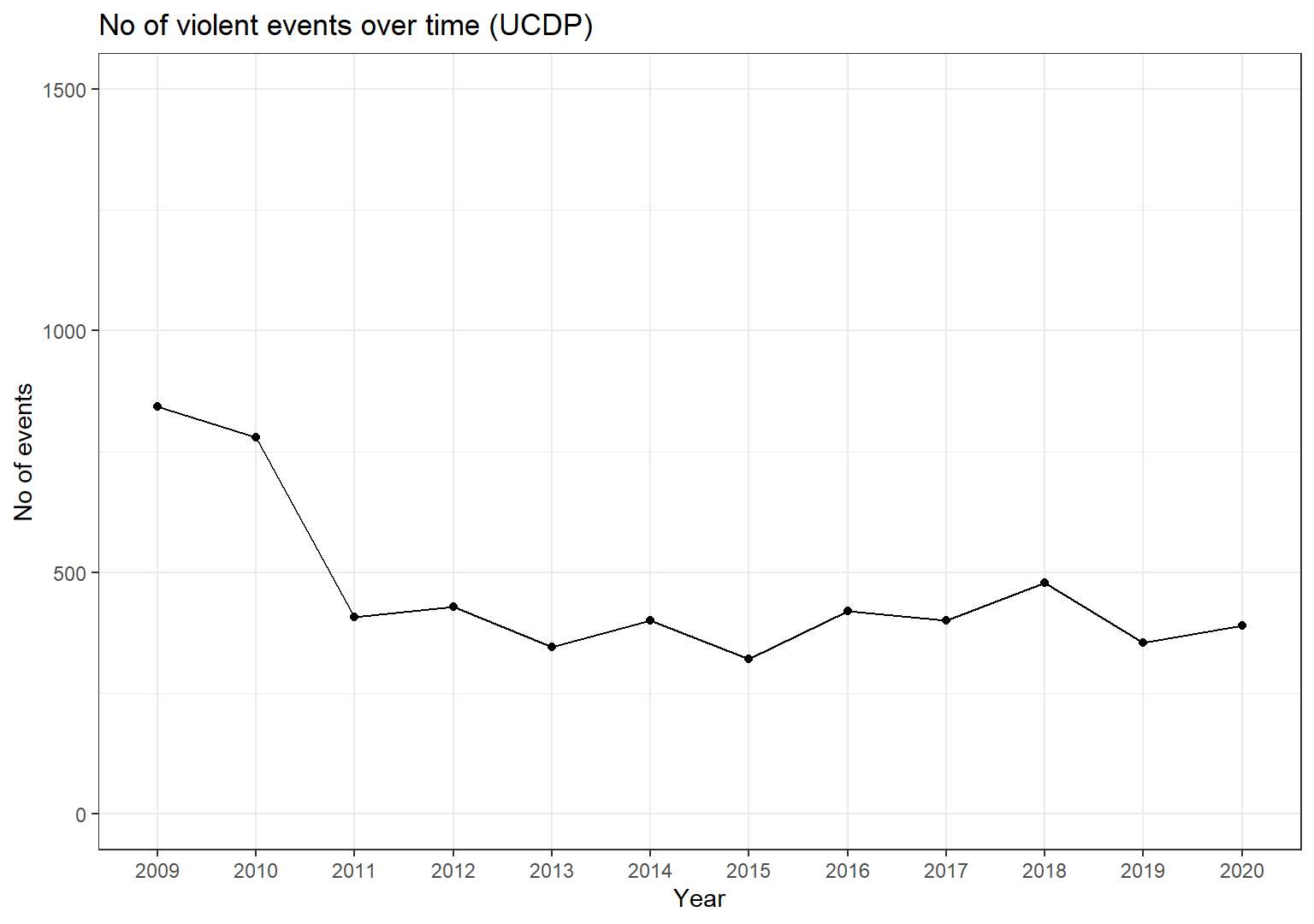

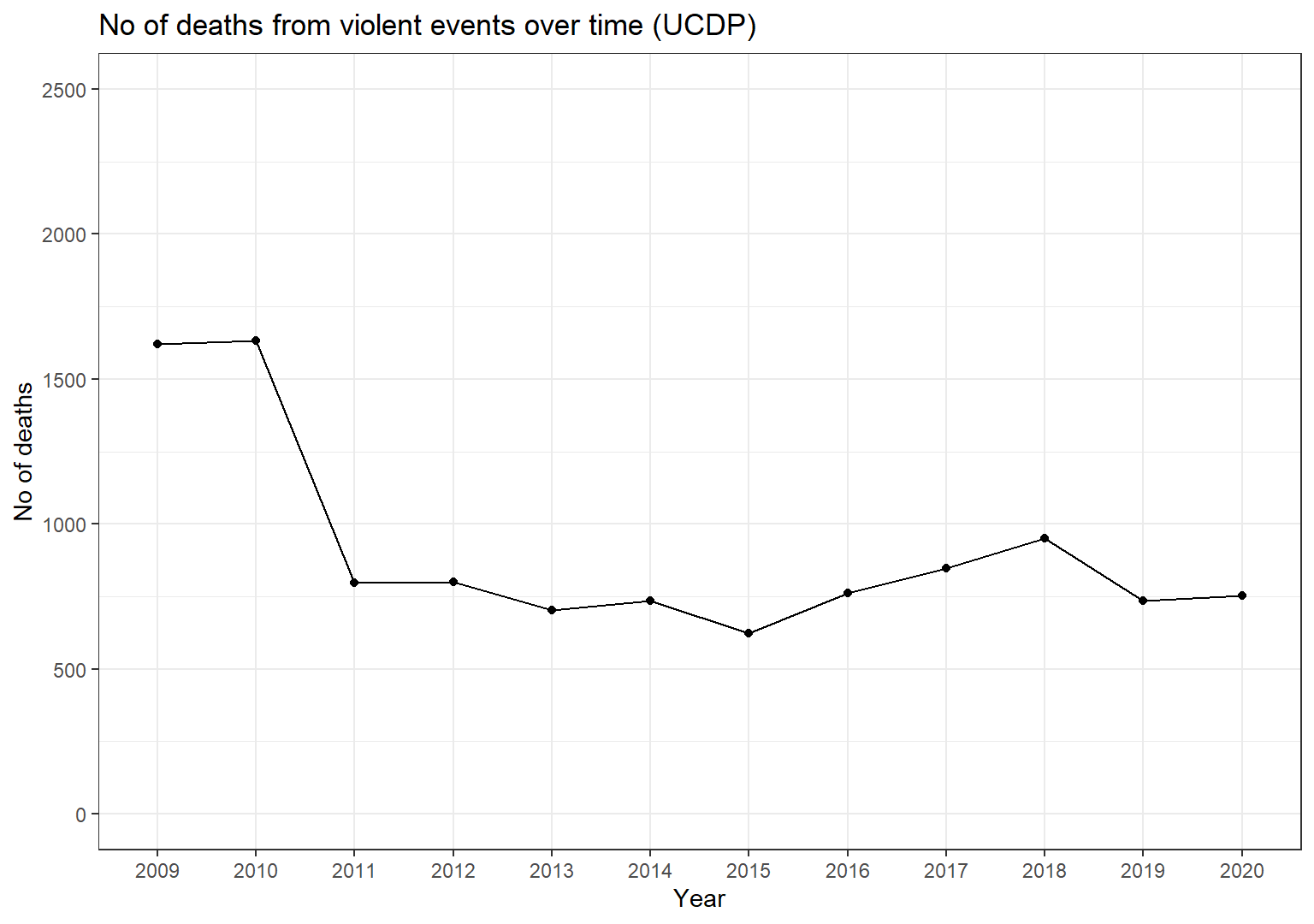


**Figure S1:** Number of high intensity conflict events and deaths by year from Uppsala Conflict Data Program’s India data (2009-2020)

**Table S2:** Multivariate regression results for the association of violence on risks of child anthropometric outcomes comparing children exposed in single and multiple developmental periods in National Family Health Survey of India-5 (NFHS) (2019-21)

| **Development period of violence exposure Binary exposure (Y or N)** | **Stunting** | | | **Underweight** | | | **Wasting** | | |
| --- | --- | --- | --- | --- | --- | --- | --- | --- | --- |
|  | ***Risk Ratio*** | ***CI*** | ***p value*** | ***Risk Ratio*** | ***CI*** | ***p value*** | ***Risk Ratio*** | ***CI*** | ***p value*** |
| In-utero exposure only (in children <= 5 years) | 1.06 | *1.00, 1.12* | *0.06* | 1.12 | *1.06, 1.2* | *0.00* | 1.02 | *0.96, 1.09* | *0.49* |
| 0-3 years only (in children <=5 years) | 1.14 | *1.09, 1.20* | *0.00* | 1.14 | *1.09, 1.2* | *0.00* | 0.99 | *0.94, 1.05* | *0.83* |
| In both periods  (in children <=5 years) | 1.21 | *1.15, 1.27* | *0.00* | 1.16 | *1.1, 1.22* | *0.00* | 1.07 | *1.01, 1.13* | *0.02* |


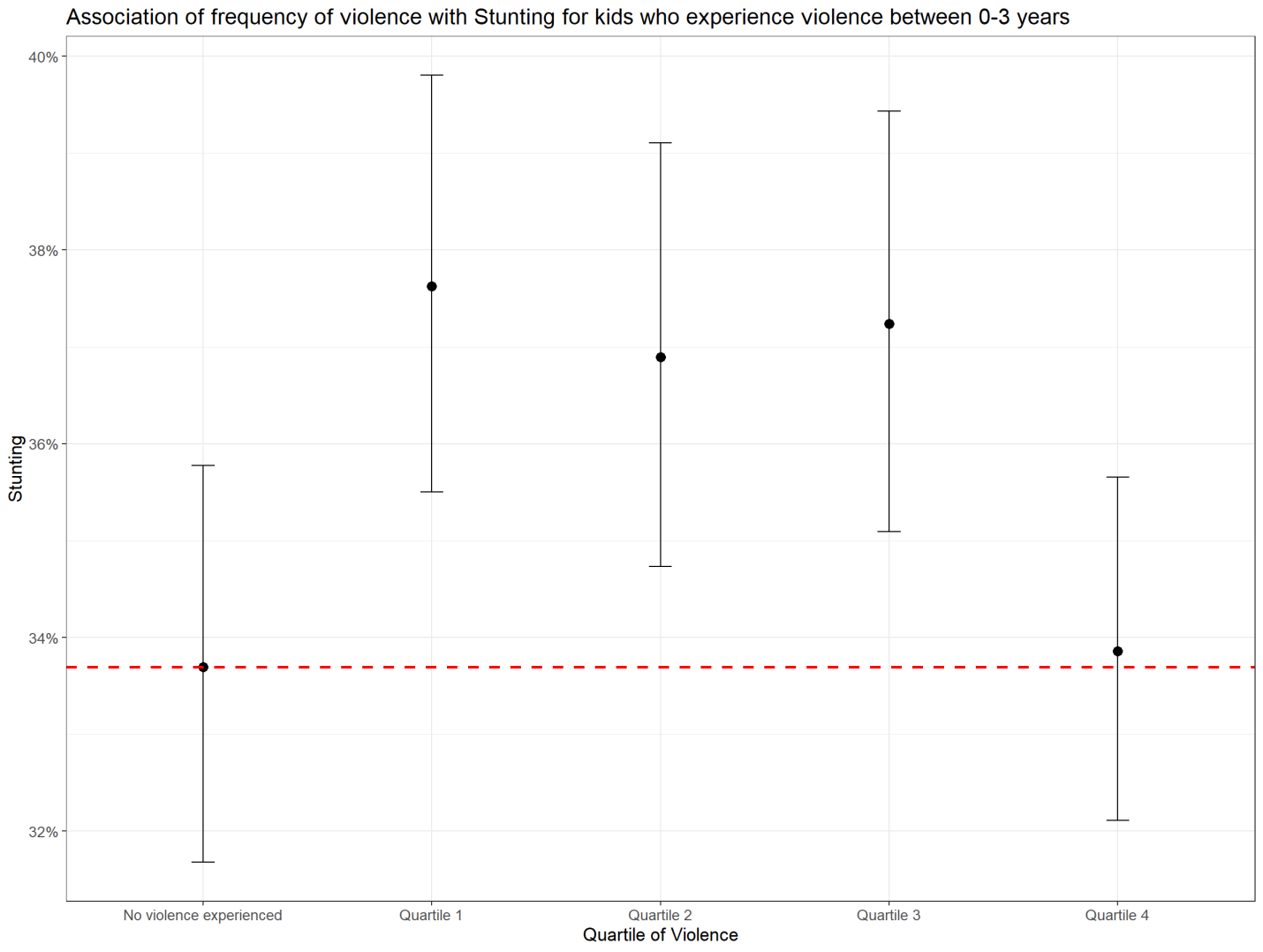


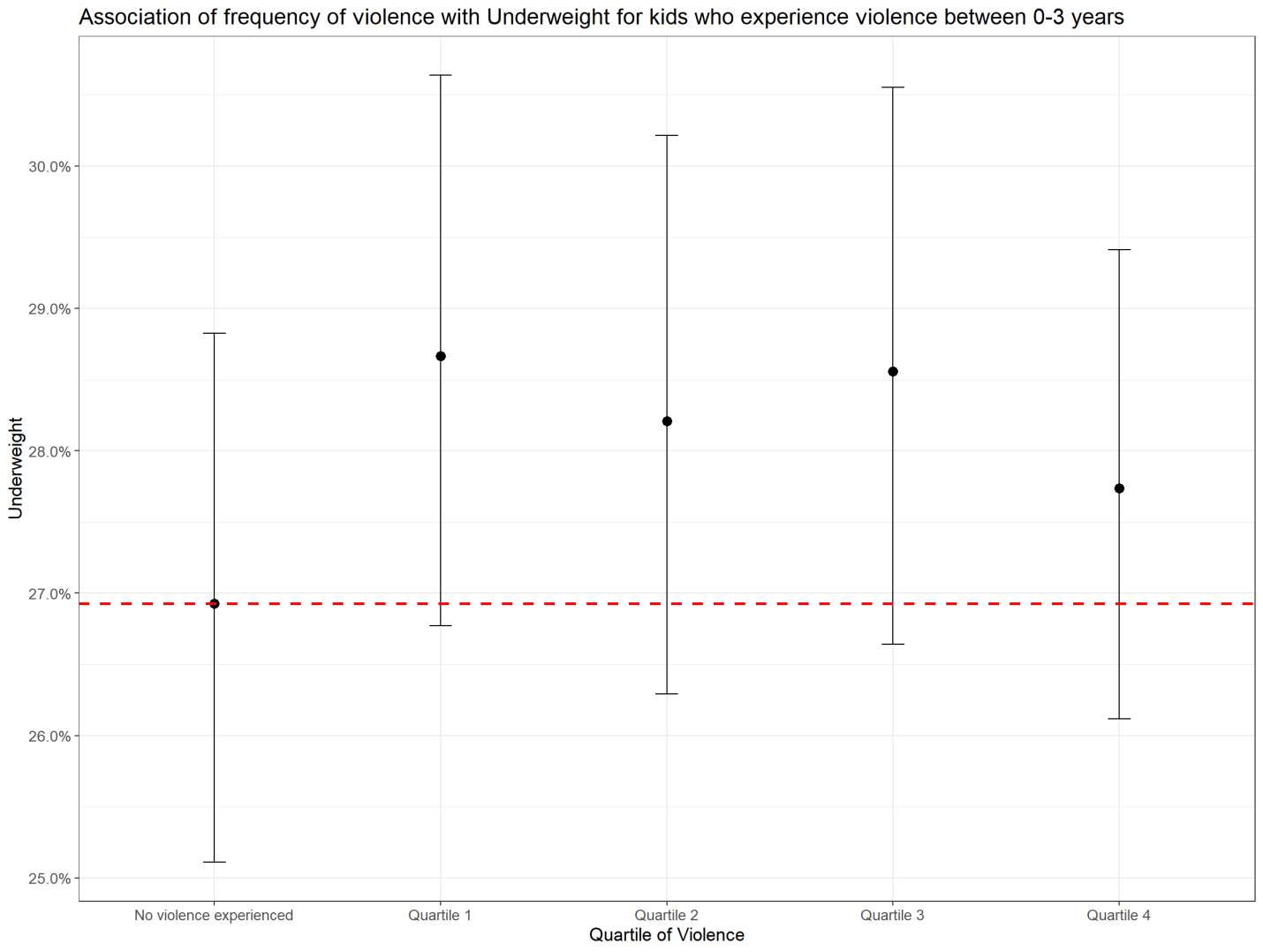


**Figure S2 (A):** Predicted prevalence of stunting and underweight nationally for quartiles of violence exposure for children 0-3 years, for National Family Health Survey-5 (2019-21). The red line indicates the predicted prevalence of underweight for children who were not exposed to conflicts


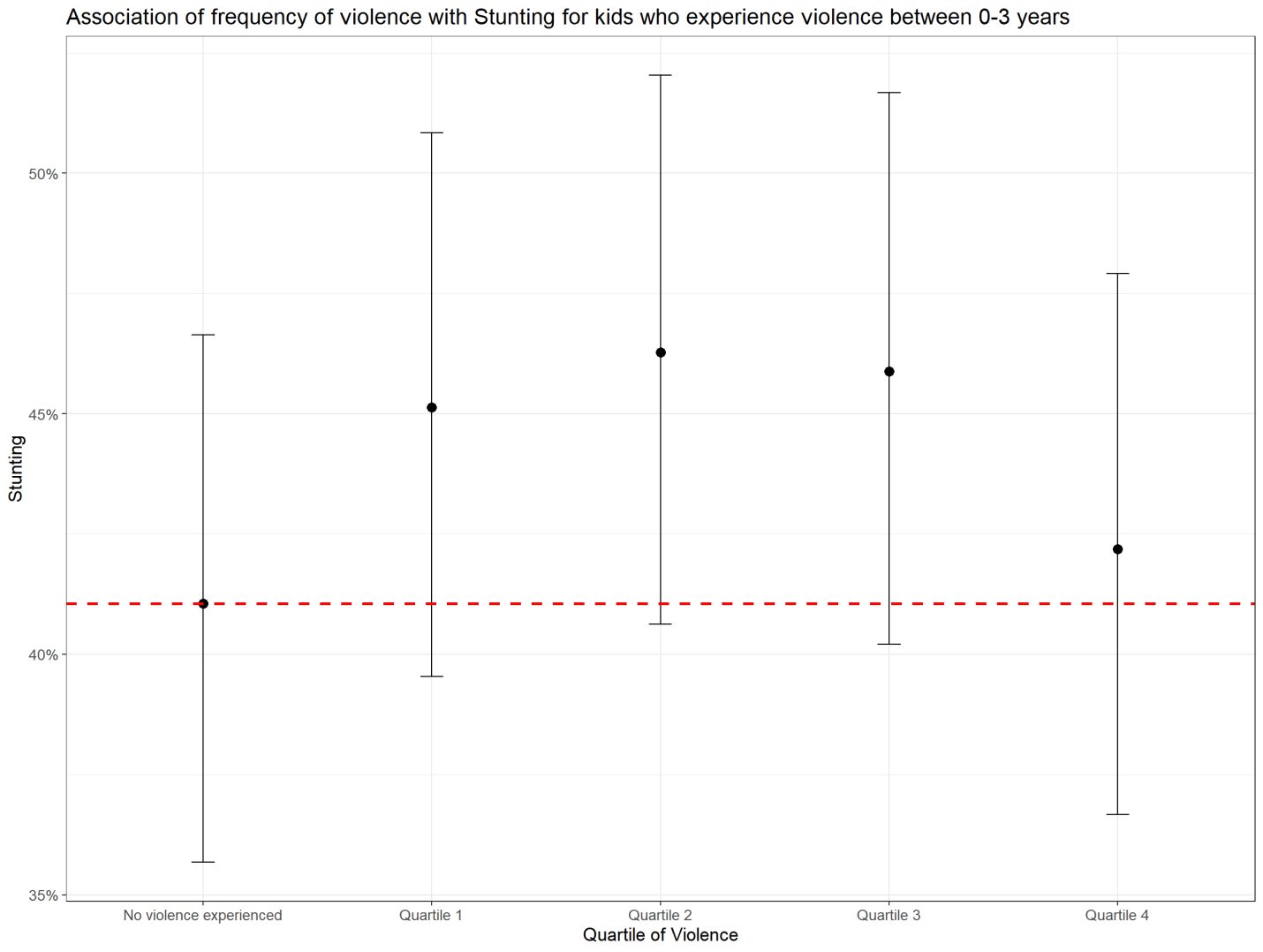


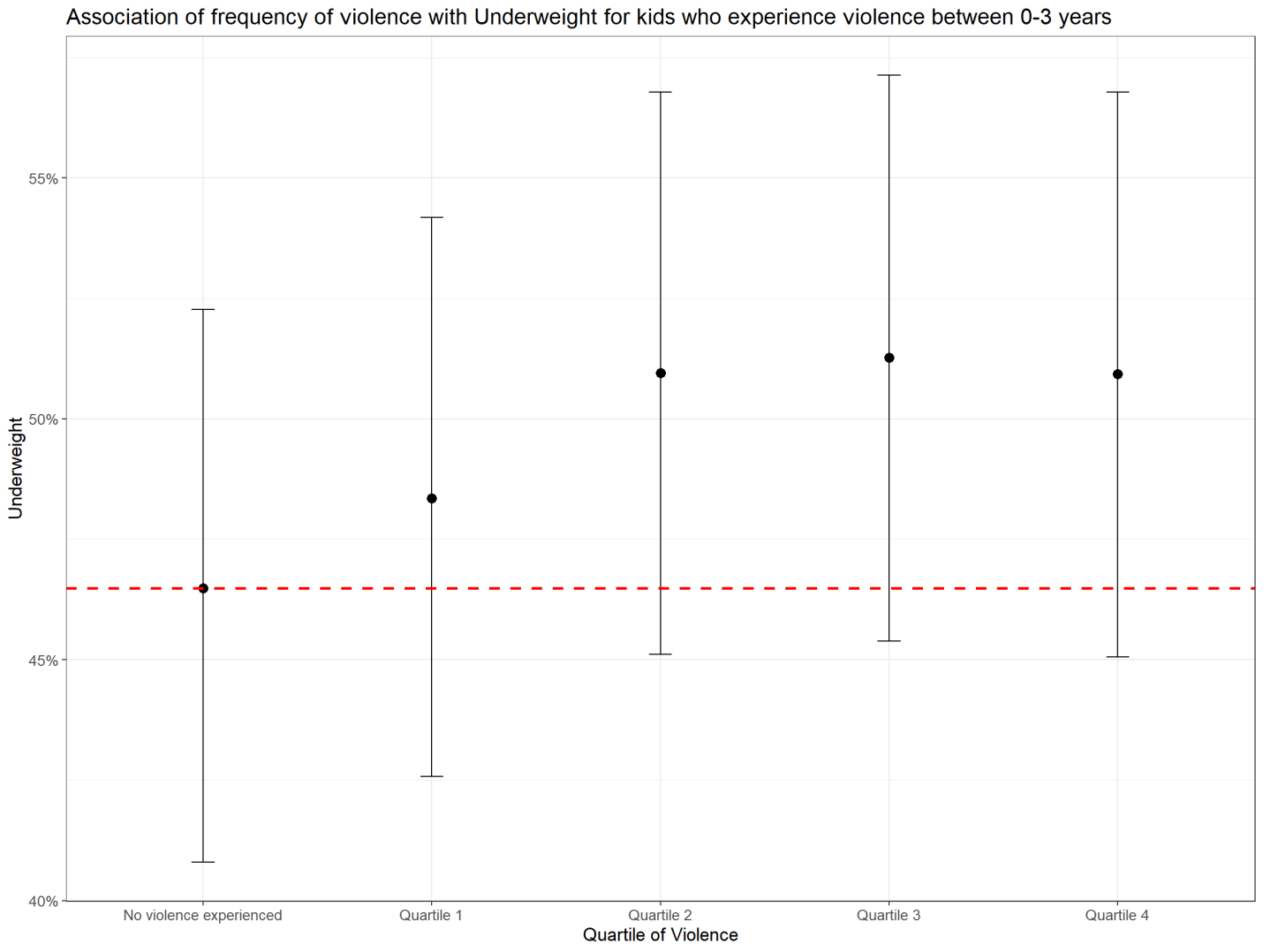


**Figure S2 (B):** Predicted prevalence of stunting and underweight nationally for quartiles of violence exposure for children 0-3 years, for National Family Health Survey-4 (2015-16). The red line indicates the predicted prevalence of underweight for children who were not exposed to conflicts


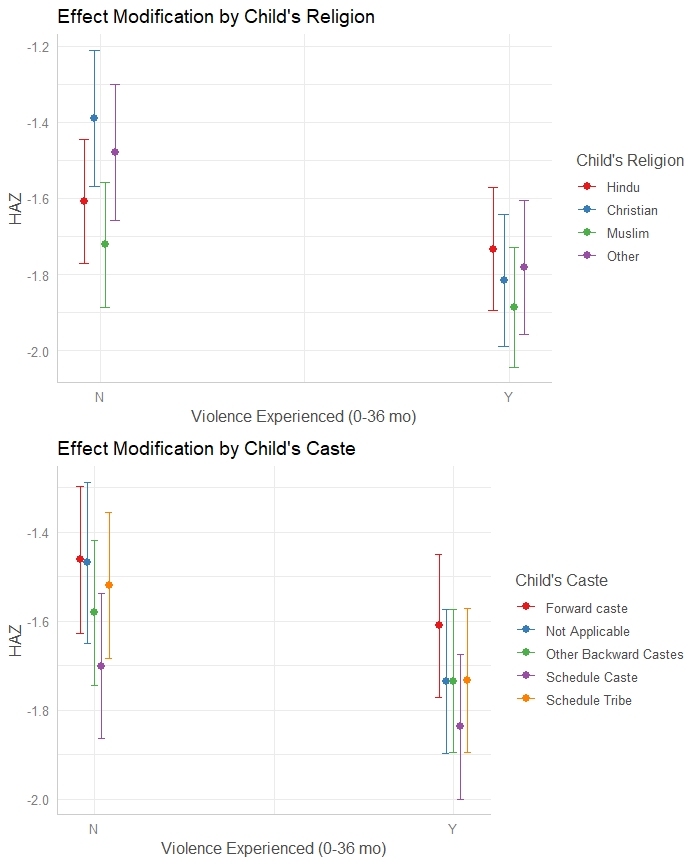


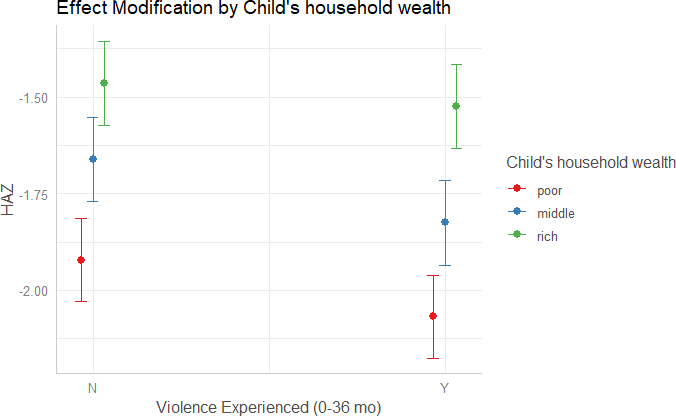


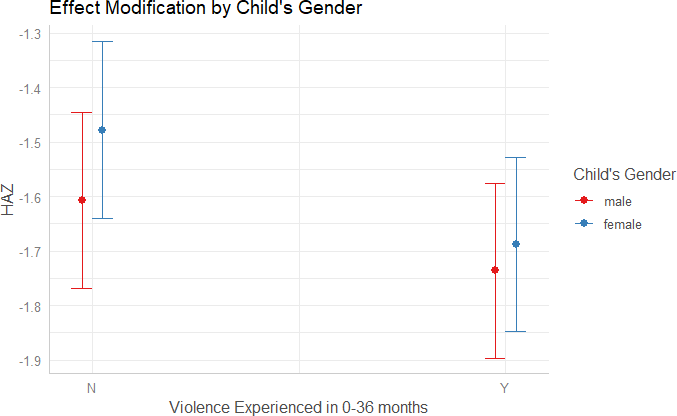


**Figure S3:** Effect measure modification by different socioeconomic characteristics in the association of violence exposure between 0-3 years of age and child Height-For-Age-Z-scores, National Family Health Survey (2019-21)


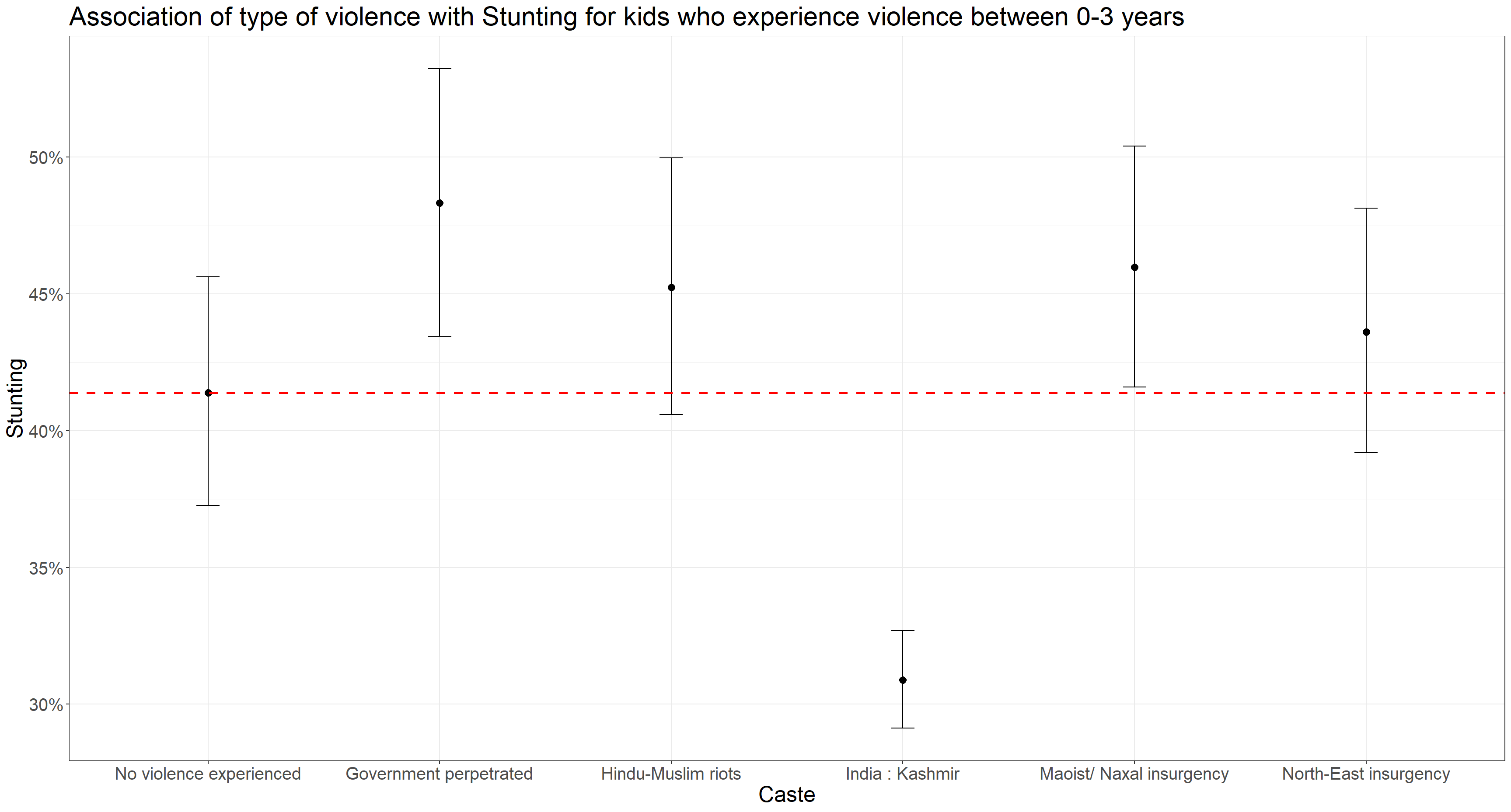


**Figure S4:** Effect measure modification by types of chronic violence, in the association of exposure to conflicts in 0-3 years, and predicted probabilities of stunting in children <=5 years, as an example. Here the red line indicates predicted prevalence of stunting under no violence exposure, which is the reference group.

**Table S3:** Multivariate regression results for association between in-utero violence exposure and birth weight in children < =5 years in National Family Health Surveys (NFHS) 2015-16 and 2019-21

|  | **NFHS 4** | | | **NFHS 5** | | |
| --- | --- | --- | --- | --- | --- | --- |
| **Low Birthweight** | ***Risk Ratio*** | ***95% CI*** | ***p value*** | ***Risk Ratio*** | ***95% CI*** | ***p value*** |
|  |  |  |  |  |  |  |
| In-utero exposure | 1.07 | *1.04, 1,10* | *0.00* | 1.06 | *1.03, 1.10* | *0.00* |
| (in children <= 5 years) |  |  |  |  |  |  |

**Table S4:** Multivariate regression results for association between violence exposure on maternal and childhood anemia using National Family Health Surveys (NFHS) 2015-16 and 2019-21

| **Children's experience of violence** | **Anemia ( Hemoglobin < 11 g/DL)** | | |
| --- | --- | --- | --- |
|  | **Risk Ratio** | **95% CI** | **p value** |
| Exposure during 0-3 years | 1.1 | 1.06, 1.13 | 0.00 |
| **Mother's experience of violence during last two years** | **Risk Ratio** | **95% CI** | **p value I** |
| Binary exposure (exposed or not) | 1.09 | 1.06, 1.13 | 0.00 |
| Quartile 2 | 1.07 | 1.01, 1.12 | 0.01 |
| Quartile 3 | 1.13 | 1.07,1.19 | 0.00 |
| Quartile 4 | 1.25 | 1.18 ,1.32 | 0.00 |

**Table S5:** Multivariate regression results for association between in-utero violence exposure and anthropometric outcomes in children < 2 years, who had at least one sibling who did not experience violence. Data: National Family Health Surveys (NFHS) 2015-16 and 2019-21

|  | **HAZ** | | | **Stunting** | | |
| --- | --- | --- | --- | --- | --- | --- |
| **Sibling Model** | ***Estimate (Mean difference)*** | ***95% CI*** | ***p value*** | ***Risk Ratio*** | ***95% CI*** | ***p value*** |
|  |  |  |  |  |  |  |
| Development period : In-utero exposure  (in children <= 3 years) | -0.24 | *-0.33, -0.16* | *0.00* | 1.19 | *1.10, 1.29* | *0.00* |

**Table S6: Multivariate regression results for the association of violence exposure on child anthropometric Z- scores nationally in states which were almost entirely(92%( sampled pre COVID National Family Health Survey (2019-21)**

*Included states/UTs: Kerala, Jammu and Kashmir, Goa, Ladakh, West Bengal, Mizoram, Lakshwadeep, Assam, Dadra and Nagar Haveli and Daman and Diu, Maharahstra, Himachal Pradesh, Meghalaya, Gujarat, Telangana, Manipur, Tripura, Nagaland, Karnataka, Sikkim, Andhra Pradesh, Andaman and Nicobar Islands and Bihar.

| **Development period of violence exposure Binary exposure (Y or N)** | **Stunting** | | | **Underweight** | | | **Wasting** | | |
| --- | --- | --- | --- | --- | --- | --- | --- | --- | --- |
|  | ***Risk Ratio*** | ***95% CI*** | ***p value*** | ***Risk Ratio*** | ***95% CI*** | ***p value*** | ***Risk Ratio*** | ***95% CI*** | ***p value*** |
| In-utero exposure  (in children <= 2 years) | 1.21 | *1.12, 1.31* | *0.00* | 1.11 | *1.02, 1.19* | *0.01* | 0.95 | *0.87, 1.03* | *0.2* |
| 0-3 years (in children <=5 years) | 1.12 | *1.06, 1.18* | *0.00* | 1.09 | *1.03, 1.15* | *0.00* | 1.075 | *1.01, 1.14* | *0.02* |

| **Development period of violence exposure Binary exposure (Y or N)** | **HAZ** | | | **WAZ** | | | **WHZ** | | |
| --- | --- | --- | --- | --- | --- | --- | --- | --- | --- |
|  | **Mean Estimate** | **95% CI** | **p value** | **Mean Estimate** | **95% CI** | **p value** | **Mean Estimate** | **95% CI** | **p value** |
| In-utero exposure  (in children <= 2 years) | -0.14 | *-0.21, -0.06* | *0.00* | -0.07 | *-0.12, -0.02* | *0.01* | 0.06 | *0, 0.13* | *0.01* |
| 0-3 years (in children <=5 years) | -0.04 | *-0.08, 0.01* | *0.04* | -0.05 | *-0.09, -0.02* | *0.00* | -0.09 | *-0.13, -0.05* | *-0.00* |

**Table S7:** Multivariate regression results for the association of violence exposure on child anthropometric Z- scores nationally in states which sampled both pre and post COVID, National Family Health Survey (2019-21)

| **Development period of violence exposure Binary exposure (Y or N)** | **Stunting** | | | **Underweight** | | | **Wasting** | | |
| --- | --- | --- | --- | --- | --- | --- | --- | --- | --- |
|  | ***Risk Ratio*** | ***95% CI*** | ***p value*** | ***Risk Ratio*** | ***95% CI*** | ***p value*** | ***Risk Ratio*** | ***95% CI*** | ***p value*** |
| In-utero exposure  (in children <= 2 years) | 1.04 | *0.91, 1.08* | *0.86* | 1.04 | *0.96, 1.13* | *0.36* | 1.05 | *0.95, 1.15* | *0.33* |
| 0-3 years (in children <=5 years) | 1.21 | *1.15, 1.28* | *0.00* | 1.06 | *1.00, 1.12* | *0.04* | 0.905 | *0.85, 0.97* | *0.00* |

| **Development period of violence exposure Binary exposure (Y or N)** | **HAZ** | | | **WAZ** | | | **WHZ** | | |
| --- | --- | --- | --- | --- | --- | --- | --- | --- | --- |
|  | **Mean Estimate** | **95% CI** | **p value** | **Mean Estimate** | **95% CI** | **p value** | **Mean Estimate** | **95% CI** | **p value** |
| In-utero exposure  (in children <= 2 years) | 0.00 | *-0.07, 0.08* | *0.94* | -0.03 | *-0.08, 0.02* | *0.28* | -0.02 | *-0.08, 0.04* | *0.55* |
| 0-3 years (in children <=5 years) | -0.21 | *-0.25, -0.16* | *0.00* | -0.05 | *-0.08, 0.02* | *0.00* | -0.02 | *-0.06, 0.02* | *-0.07* |

* To differentiate children who were sampled pre and post Covid lockdown in these states, we included a binary pre/post Covid flag in the regression

*Included list of states/UTs: Rajasthan, Delhi, Puducherry, Punjab, Arunachal Pradesh, Uttarakhand, Uttar Pradesh, Chattisgarh, Odisha,Madhya Pradesh, Tamil Nadu, Jharkhand, Haryana

**Table S8:** Estimating influence of distance thresholds from site of conflict on association of conflict exposure and child anthropometry (with HAZ as a sample)

| **Distance cut-off** | **Mean Estimate** | **95% CI** | **p value** |
| --- | --- | --- | --- |
| 50 km | -0.17 | -0.20, -0.14 | 0.00 |
| 75 km | -0.22 | -0.25, -0.19 | 0.00 |
| 100 km | -0.25 | -0.28,- 0.22 | 0.00 |
| 200 km | -0.19 | -0.22 ,-0.17 | 0.00 |
| 300 km | -0.23 | -0.25,-0.20 | 0.00 |


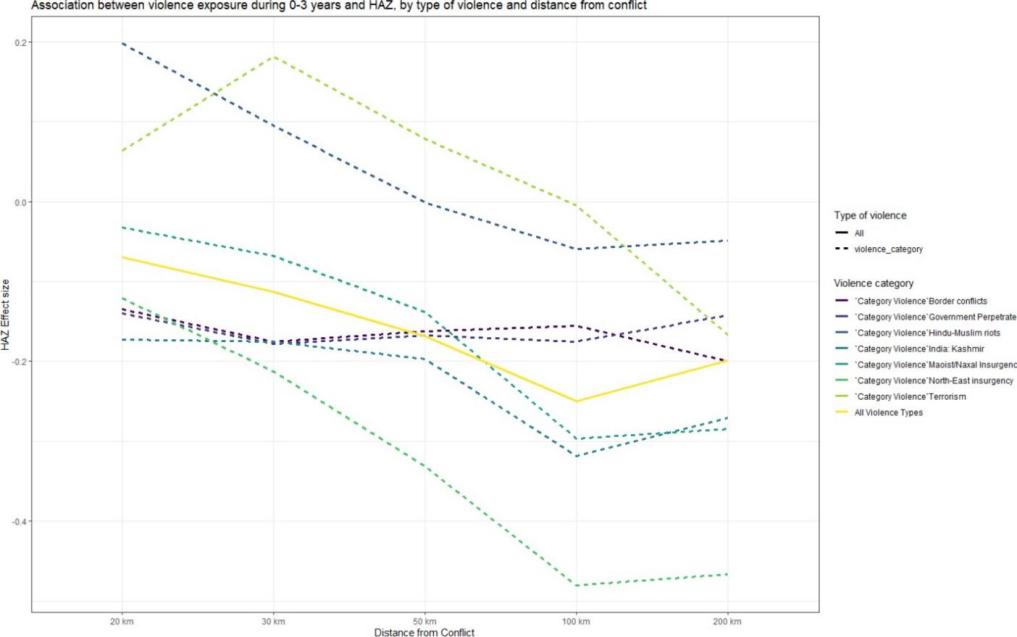


**Figure S4:** Association between violence exposure between 0-3 years and HAZ by type of violence and distance from conflict

**Table S9:** National number and percentage of children sampled by month of their height and weight measurement in National Family Health Survey (2019-21)

| **Month** | **No of children surveyed** | **% of Total** | **Cumulative %** |
| --- | --- | --- | --- |
| 6/1/2019 | 175 | 0% | 0% |
| 7/1/2019 | 15350 | 7% | 7% |
| 8/1/2019 | 20258 | 9% | 15% |
| 9/1/2019 | 24582 | 10% | 26% |
| 10/1/2019 | 21830 | 9% | 35% |
| 11/1/2019 | 14300 | 6% | 41% |
| 12/1/2019 | 4710 | 2% | 43% |
| 1/1/2020 | 13713 | 6% | 49% |
| 2/1/2020 | 23272 | 10% | 59% |
| 3/1/2020 | 13479 | 6% | 64% |
| **4/1/2020** | **2** | **0%** | **64%** |
| **10/1/2020** | **2** | **0%** | **64%** |
| 11/1/2020 | 662 | 0% | 65% |
| 12/1/2020 | 13003 | 6% | 70% |
| 1/1/2021 | 19084 | 8% | 78% |
| 2/1/2021 | 13650 | 6% | 84% |
| 3/1/2021 | 14183 | 6% | 90% |
| 4/1/2021 | 8962 | 4% | 94% |
| 5/1/2021 | 46 | 0% | 94% |
|  | 13892 | 6% | 100% |
| Total | **235155** | 100% |  |

*Row highlighted in bold indicates month with drop in sampled children

**Table S10:** Distribution of sampled children and the percentage of children sampled pre-COVID by States.

Here: Pre-covid cut-off has been defined as pre 3/25/2020, based on distributions of sampled children in Table 1 and Figure 1.

| **State Name** | **Pre-Covid** | **Post-Covid** | **Date of measurement missing** | **% Pre-Covid** |
| --- | --- | --- | --- | --- |
| Kerala | 2692 | 0 | 71 | 97% |
| Jammu & Kashmir | 5715 | 0 | 179 | 97% |
| Goa | 362 | 0 | 12 | 97% |
| Ladakh | 515 | 0 | 19 | 96% |
| West Bengal | 5436 | 0 | 232 | 96% |
| Mizoram | 2380 | 0 | 103 | 96% |
| Lakshadweep | 265 | 0 | 13 | 95% |
| Assam | 10248 | 0 | 507 | 95% |
| Dadra & Nagar Haveli And Daman & Diu | 764 | 0 | 38 | 95% |
| Maharashtra | 9123 | 0 | 475 | 95% |
| Himachal Pradesh | 2522 | 0 | 133 | 95% |
| Meghalaya | 6352 | 0 | 340 | 95% |
| Gujarat | 9434 | 0 | 521 | 95% |
| Telangana | 6998 | 0 | 394 | 95% |
| Manipur | 3085 | 0 | 176 | 95% |
| Tripura | 1980 | 0 | 118 | 94% |
| Nagaland | 2901 | 0 | 177 | 94% |
| Karnataka | 7956 | 0 | 502 | 94% |
| Sikkim | 591 | 0 | 38 | 94% |
| Andhra Pradesh | 2688 | 0 | 174 | 94% |
| Andaman & Nicobar Islands | 432 | 0 | 32 | 93% |
| Bihar | 19556 | 0 | 1731 | 92% |
| Rajasthan | 10759 | 3225 | 813 | 73% |
| Nct Of Delhi | 1647 | 1183 | 136 | 56% |
| Puducherry | 376 | 362 | 34 | 49% |
| Punjab | 2441 | 2887 | 350 | 43% |
| Arunachal Pradesh | 2197 | 3152 | 258 | 39% |
| Uttarakhand | 1482 | 2140 | 193 | 39% |
| Uttar Pradesh | 13609 | 19795 | 2709 | 38% |
| Chhattisgarh | 2824 | 5218 | 542 | 33% |
| Odisha | 2730 | 5376 | 496 | 32% |
| Madhya Pradesh | 5013 | 10343 | 1076 | 31% |
| Tamil Nadu | 1921 | 4345 | 281 | 29% |
| Jharkhand | 2919 | 6593 | 607 | 29% |
| Haryana | 1749 | 4813 | 402 | 25% |
| Chandigarh * | 0 | 169 | 10 | 0% |
| **Total** | **151662** | **69601** | **13892** | **64%** |

*Chandigarh has all children sampled in the post-covid period

**Table S 11:** Unadjusted prevalence of anthropometric outcomes in states sampled almost entirely in the pre-COVID lockdown phase, and states which sampled children both pre and post COVID lockdown phases

|  | **Anthropometric measurement period** | | |
| --- | --- | --- | --- |
| **Metric** | **Pre-COVID sample** | **Pre and post-COVID sample** | **p value** |
| n | 49667 | 69432 |  |
| Stunted (mean (SD)) | 0.34 (0.47) | 0.34 (0.47) | 0.712 |
| Underweight (mean (SD)) | 0.28 (0.45) | 0.30 (0.46) | <0.001 |
| Wasted (mean (SD)) | 0.16 (0.37) | 0.16 (0.37) | 0.08 |
| Severe Stunted (mean (SD)) | 0.15 (0.36) | 0.14 (0.35) | <0.001 |
| Severe Underweight (mean (SD)) | 0.10 (0.30) | 0.10 (0.30) | 0.514 |
| Severe Wasted (mean (SD)) | 0.07 (0.26) | 0.06 (0.24) | <0.001 |


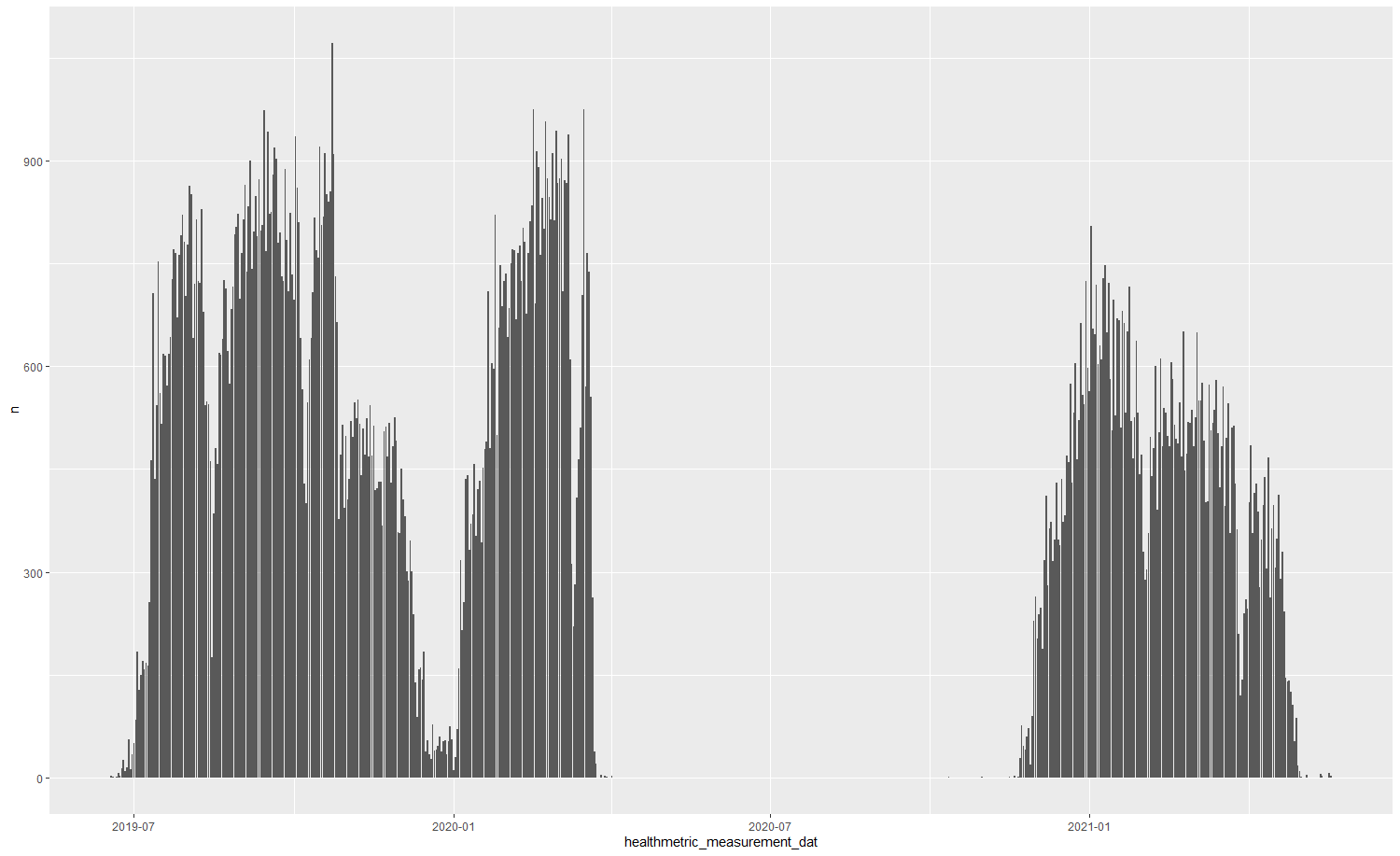


**Figure S5:** Trends in month/year of height and weight measurement of sampled children in NFHS 5 (2019-21)

**Table S12:** Multivariate regression results for the association of violence exposure on child anthropometric Z- scores nationally (distance cut-off: 100 km) in National Family Health Surveys (2015-16)

| **Development period of violence exposure Binary Exposure (Y or N)** | **HAZ** | | | **WAZ** | | | **WHZ** | | |
| --- | --- | --- | --- | --- | --- | --- | --- | --- | --- |
|  | **Mean Estimate** | ***95% CI*** | ***p value*** | **Mean Estimate** | ***95% CI*** | ***p value*** | **Mean Estimate** | ***95% CI*** | ***p value*** |
| In-utero exposure (in children <= 2 years) | -0.11 | *-0.15, -0.07* | 0.00 | -0.04 | *-0.06, -0.01* | *0.00* | 0.04 | *0.01, 0.07* | *0.01* |
| 0-3 years | -0.2 | *-0.22, -0.18* | 0.00 | -0.07 | *-0.09, -0.05* | *0.00* | -0.01 | *-0.03, 0.01* | *0.22* |

**Table S 13: Comparison of effect sizes of HAZ in the present study with other studies**


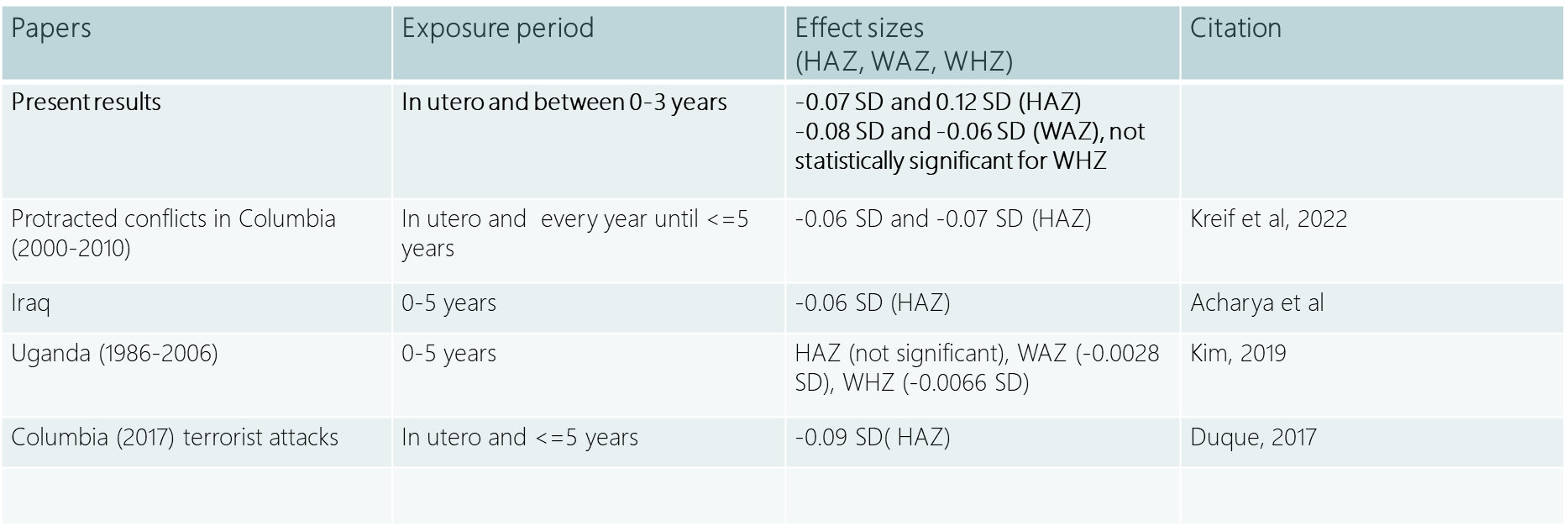


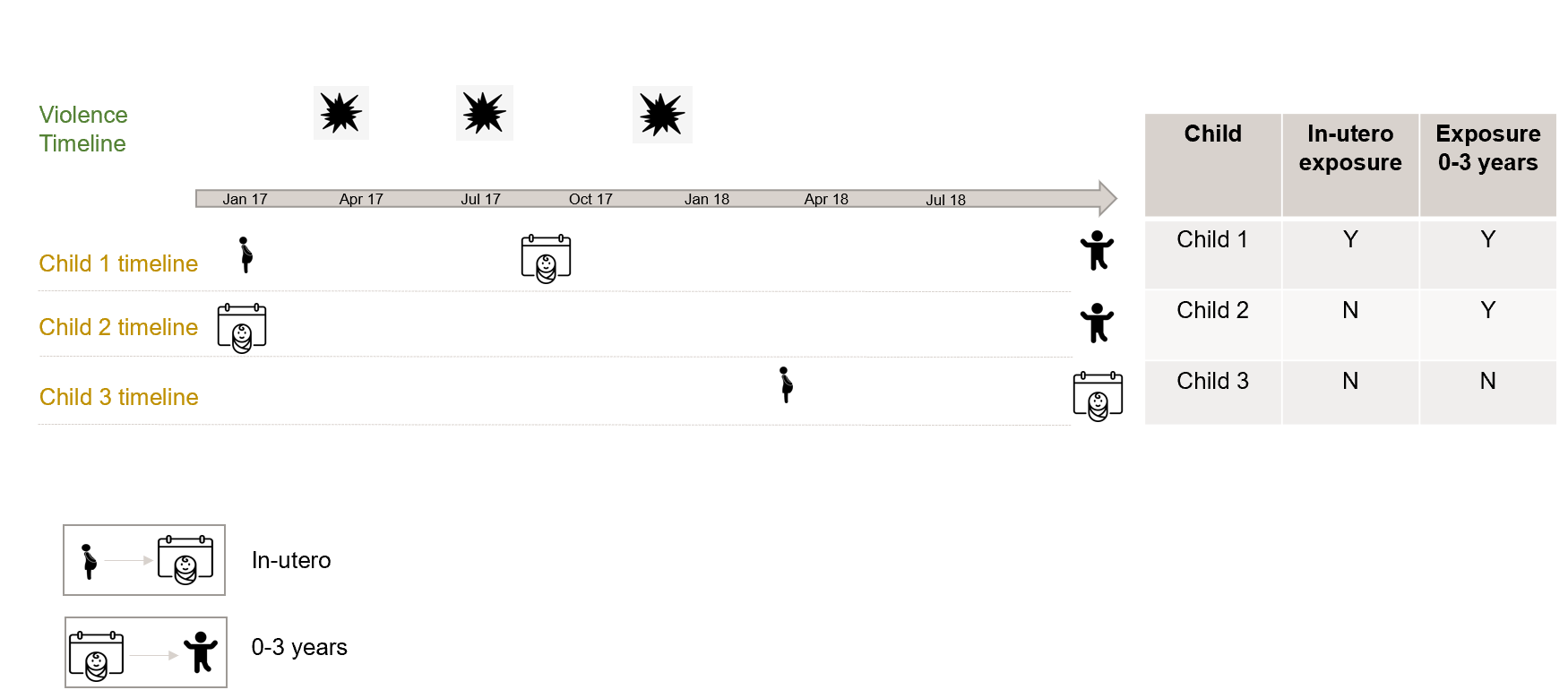


**Figure S6: Flow chart explaining study design with three illustrative children in sample.** Here the flashy sparks indicate violent events. Child 1 experiences violence both in-utero and during 0-3 years, child 2 is born in January 17, before any violent events, so is treated as unexposed for in-utero exposures, and exposed for early childhood exposures. Child 3 who is conceived after violent events, is treated as unexposed for both developmental periods. This highlights two limitations in our design which possibly underestimated exposure to violence- 1)all pregnancies were treated as full term, 2)pre-conception exposures were not accounted for.
